# Genomic analyses implicate hormonal and metabolic dysregulation in polycystic ovary syndrome

**DOI:** 10.1038/s41588-026-02543-9

**Published:** 2026-04-23

**Authors:** Loes M. E. Moolhuijsen, Jia Zhu, Benjamin H. Mullin, Natàlia Pujol-Gualdo, Ky’Era V. Actkins, Jasmine A. Mack, Hridya Rao, Bhavi Trivedi, Katherine A. Kentistou, Yajie Zhao, David Westergaard, Jaakko S. Tyrmi, Gudmar Thorleifsson, Yanfei Zhang, Laura Wittemans, Amber DeVries, Kelly Brewer, Ryan Sisk, Rebecca Danning, Michael H. Preuss, Michelle R. Jones, Katherine S. Ruth, Marianne Andersen, Ricardo Azziz, Karina Banasik, Michael Boehnke, Linda Broer, Søren Brunak, Yee-Ming Chan, Daniel I. Chasman, Mark Daly, David A. Ehrmann, Bart C. Fauser, Lars G. Fritsche, M. Geoffrey Hayes, Chunyan He, Hongyan Huang, Irina Kowalska, Peter Kraft, Richard S. Legro, Nan Lin, Ruth J. Loos, Yvonne V. Louwers, Reedik Magi, Mark I. McCarthy, Laure Morin-Papunen, Jean V. Morrison, Cynthia Morton, Girish N. Nadkarni, Benjamin M. Neale, Henriette Svarre Nielsen, Mette Nyegaard, Sisse R. Ostrowski, Ole B. V. Pedersen, Erik Sørensen, Christina Mikkelsen, Christian Erikstrup, Kathrine A. Kaspersen, Mie T. Bruun, Bitten Aagaard, Henrik Ullum, Barbara Obermayer-Pietsch, Aarno Palotie, Mary P. Reeve, Andres Salumets, Richa Saxena, Timothy D. Spector, Bronwyn G. A. Stuckey, Unnur Thorsteinsdottir, André G. Uitterlinden, Margrit Urbanek, Sebastian Zöllner, Genes and Health Research Team, Bhavi Trivedi, David A. van Heel, DBDS Genomic Consortium, David Westergaard, Karina Banasik, Søren Brunak, Mette Nyegaard, Sisse R. Ostrowski, Ole B. V. Pedersen, Erik Sørensen, Christina Mikkelsen, Christian Erikstrup, Kathrine A. Kaspersen, Mie T. Bruun, Bitten Aagaard, Unnur Thorsteinsdottir, Henrik Ullum, Kari Stefansson, 23andMe Research Team, Adam Auton, Alan Kwong, Anjali J. Shastri, Barry Hicks, Catherine H. Weldon, David A. Hinds, Emily DelloRusso, Emily M. Rios, Joyce Y. Tung, Kahsaia de Brito, Katelyn Kukar Bond, Keng-Han Lin, Matthew H. McIntyre, Matthew J. Kmiecik, Qiaojuan Jane Su, Robert K. Bell, Sayantan Das, Shubham Saini, Stella Aslibekyan, Vinh Tran, Wanwan Xu, Alisa P. Lehman, Noura S. Abul-Husn, R. Ryanne Wu, Rebecca M. K. Berns, Ruth I. Tennen, Stacey B. Detweiler, Aditya Ambati, Anna Guan, Bertram L. Koelsch, Chris German, Éadaoin Harney, Ethan M. Jewett, G. David Poznik, James R. Ashenhurst, Jingran Wen, Peter R. Wilton, Steven J. Micheletti, William A. Freyman, David A. van Heel, Joel N. Hirschhorn, Kari Stefansson, John R. B. Perry, Unnur Styrkarsdottir, Scott G. Wilson, Terhi Piltonen, Triin Laisk, Marjo-Riitta Jarvelin, Kharis Burns, Anne E. Justice, Hannele Laivuori, Ken K. Ong, Mark O. Goodarzi, Lea K. Davis, Andrea Dunaif, Cecilia M. Lindgren, Joop S. E. Laven, Stephen Franks, Jenny A. Visser, Corrine K. Welt, Tugce Karaderi, Felix R. Day

**Affiliations:** 1 Department of Internal Medicine, Erasmus MC, University Medical Center Rotterdam, Rotterdam, the Netherlands; 2 Division of Endocrinology, Boston Children’s Hospital, Boston, MA USA; 3 Department of Pediatrics, Harvard Medical School, Boston, MA USA; 4 Program in Medical and Population Genetics, The Broad Institute of MIT and Harvard, Cambridge, MA USA; 5 Department of Endocrinology & Diabetes, Sir Charles Gairdner Hospital, Nedlands, Western Australia Australia; 6 School of Biomedical Sciences, University of Western Australia, Crawley, Western Australia Australia; 7 Estonian Genome Centre, Institute of Genomics, University of Tartu, Tartu, Estonia; 8 Department of Obstetrics and Gynecology, Research Unit of Clinical Medicine, Medical Research Centre, Oulu University Hospital, University of Oulu, Oulu, Finland; 9 Division of Genetic Medicine, Department of Medicine, Vanderbilt University Medical Center, Nashville, TN USA; 10 Vanderbilt Genetics Institute, Vanderbilt University Medical Center, Nashville, TN USA; 11 Department of Biostatistics, University of Michigan, Ann Arbor, MI USA; 12 Department of Obstetrics and Gynaecology, University of Cambridge, Cambridge, UK; 13 Biostatistics and Computational Biology Branch, National Institute of Environmental Health Sciences, National Institutes of Health, Research Triangle Park, NC USA; 14 Department of Population Health Sciences, Geisinger, Danville, PA USA; 15 Department of Biobehavioral Health, Pennsylvania State University, University Park, PA USA; 16 Blizard Institute, Barts and the London School of Medicine and Dentistry, Queen Mary University of London, London, UK; 17 MRC Epidemiology Unit, Institute of Metabolic Science, School of Clinical Medicine, University of Cambridge, Cambridge, UK; 18 Translational Disease Systems Biology, Novo Nordisk Foundation Center for Protein Research, Faculty of Health and Medical Sciences, University of Copenhagen, Copenhagen, Denmark; 19 Department Obstetrics and Gynecology, Copenhagen University Hospital Hvidovre, Hvidovre, Denmark; 20 Center for Child, Adolescent and Maternal Health Research, Faculty of Medicine and Health Technology, Tampere University, Tampere, Finland; 21 Center for Life Course Health Research, Faculty of Medicine, University of Oulu, Oulu, Finland; 22 deCODE genetics/Amgen, Reykjavik, Iceland; 23 Population Analytics and Insights, Data Science and Digital Health, Janssen R&D, Springhouse, PA USA; 24 The Wellcome Trust Centre for Human Genetics, University of Oxford, Oxford, UK; 25 Center for Bioinformatics & Functional Genomics, Department of Biomedical Sciences, Cedars-Sinai Medical Center, Los Angeles, CA USA; 26 Division of Endocrinology, Diabetes and Bone Disease, Icahn School of Medicine at Mount Sinai, New York City, NY USA; 27 Division of Cardiology, Department of Medicine, Northwestern University Feinberg School of Medicine, Chicago, IL USA; 28 Brigham and Women’s Hospital, Boston, MA USA; 29 The Charles Bronfman Institute for Personalized Medicine, Icahn School of Medicine at Mount Sinai, New York City, NY USA; 30 Department of Clinical and Biomedical Science, University of Exeter, Exeter, UK; 31 Odense University Hospital, University of Southern Denmark, Odense, Denmark; 32 Obstetrics & Gynecology, Medicine, and Healthcare Organization & Policy, Schools of Medicine and Public Health, University of Alabama at Birmingham, Birmingham, AL USA; 33 Center for Statistical Genetics, Department of Biostatistics, University of Michigan, Ann Arbor, MI USA; 34 Department of Public Health, Faculty of Health and Medical Sciences, University of Copenhagen, Copenhagen, Denmark; 35 Harvard Medical School, Boston, MA USA; 36 Broad Institute of MIT and Harvard and Massachusetts General Hospital, Harvard Medical School, Boston, MA USA; 37 Institute for Molecular Medicine Finland (FIMM), Helsinki Institute of Life Science, University of Helsinki, Helsinki, Finland; 38 Analytical and Translational Genetics Unit, Massachusetts General Hospital, Boston, MA USA; 39 Department of Medicine, Section of Adult and Paediatric Endocrinology, Diabetes, and Metabolism, The University of Chicago, Chicago, IL USA; 40 Department of Reproductive Medicine and Gynaecology, University of Utrecht & University Medical Center, Utrecht, the Netherlands; 41 Division of Endocrinology, Metabolism, and Molecular Medicine, Department of Medicine, Northwestern University Feinberg School of Medicine, Chicago, IL USA; 42 Department of Anthropology, Northwestern University, Evanston, IL USA; 43 Center for Genetic Medicine, Northwestern University Feinberg School of Medicine, Chicago, IL USA; 44 Department of Obstetrics and Gynecology, Brigham and Women’s Hospital, Boston, MA USA; 45 Departments of Epidemiology and Biostatistics, Harvard T.H. Chan School of Public Health, Boston, MA USA; 46 Department of Internal Medicine and Metabolic Diseases, Medical University of Białystok, Białystok, Poland; 47 Department of Obstetrics and Gynecology and Public Health Sciences, Penn State University College of Medicine, Hershey, PA USA; 48 The Genetics of Obesity and Related Metabolic Traits Program, Icahn School of Medicine at Mount Sinai, New York City, NY USA; 49 The Mindich Child Health and Development Institute, Icahn School of Medicine at Mount Sinai, New York City, NY USA; 50 The Novo Nordisk Foundation Center for Basic Metabolic Research, Faculty of Health and Medicine, University of Copenhagen, Copenhagen, Denmark; 51 Division of Reproductive Endocrinology and Infertility, Department of Obstetrics and Gynaecology, Erasmus MC, University Medical Center, Rotterdam, the Netherlands; 52 Oxford Centre for Diabetes, Endocrinology and Metabolism, University of Oxford, Oxford, UK; 53 Oxford NIHR Biomedical Research Centre, Churchill Hospital, Oxford, UK; 54 Research Unit of Clinical Medicine, University of Oulu, Oulu, Finland; 55 Department of Human Genetics, University of Chicago, Chicago, IL USA; 56 The Developmental Genome Anatomy Project, Brigham and Women’s Hospital, Boston, MA USA; 57 Department of Obstetrics, Gynecology and Reproductive Biology, Brigham and Women’s Hospital, Boston, MA USA; 58 Department of Pathology, Brigham and Women’s Hospital, Boston, MA USA; 59 Icahn School of Medicine at Mount Sinai, New York City, NY USA; 60 Stanley Center for Psychiatric Research, Broad Institute of MIT and Harvard, Cambridge, MA USA; 61 Analytic and Translational Genetics Unit, Massachusetts General Hospital and Harvard Medical School, Boston, MA USA; 62 Institute of Clinical Medicine, University of Copenhagen, Copenhagen, Denmark; 63 Department of Clinical Medicine, Faculty of Health and Medical Sciences, University of Copenhagen, Copenhagen, Denmark; 64 Department of Health Science and Technology, Aalborg University, Gistrup, Denmark; 65 Department of Clinical Immunology, Rigshospitalet, University of Copenhagen, Copenhagen, Denmark; 66 Department of Clinical Immunology, Zealand University Hospital, Køge, Denmark; 67 Novo Nordisk Foundation Center for Basic Metabolic Research, Faculty of Health and Medical Science, Copenhagen University, Copenhagen, Denmark; 68 Department of Clinical Immunology, Aarhus University Hospital, Aarhus, Denmark; 69 Department of Clinical Medicine, Aarhus University, Aarhus, Denmark; 70 Danish Big Data Centre for Environment and Health (BERTHA), Aarhus University, Aarhus, Denmark; 71 Clinical Immunology Research Unit, Department of Clinical Immunology, Odense University Hospital, Odense, Denmark; 72 Department of Clinical Research, University of Southern Denmark, Odense, Denmark; 73 Department of Clinical Immunology, Aalborg University Hospital, Aalborg, Denmark; 74 Statens Serum Institut, Copenhagen, Denmark; 75 Division of Endocrinology and Diabetology, Department of Internal Medicine, Medical University of Graz, Graz, Austria; 76 Competence Centre on Health Technologies, Tartu, Estonia; 77 Department of Obstetrics and Gynecology, Institute of Clinical Medicine, University of Tartu, Tartu, Estonia; 78 Division of Obstetrics and Gynecology, Department of Clinical Science, Intervention and Technology, Karolinska Institutet and Karolinska University Hospital, Huddinge, Stockholm, Sweden; 79 Department of Twin Research and Genetic Epidemiology, King’s College London, London, UK; 80 Medical School, University of Western Australia, Crawley, Western Australia Australia; 81 Keogh Institute for Medical Research, Nedlands, Western Australia Australia; 82 Faculty of Medicine, University of Iceland, Reykjavik, Iceland; 83 Department of Epidemiology, Erasmus MC, University Medical Center, Rotterdam, the Netherlands; 84 Department of Biostatistics and Department of Psychiatry, University of Michigan, Ann Arbor, MI USA; 85 Programs in Metabolism and Medical and Population Genetics, The Broad Institute of MIT and Harvard, Cambridge, MA USA; 86 Department of Genetics, Harvard Medical School, Boston, MA USA; 87 Metabolic Research Laboratory, Wellcome-MRC Institute of Metabolic Science, School of Clinical Medicine, University of Cambridge, Cambridge, UK; 88 MRC Centre for Environment and Health, Department of Epidemiology and Biostatistics, School of Public Health, Imperial College London, London, UK; 89 Research Unit of Population Health, Faculty of Medicine, University of Oulu, Oulu, Finland; 90 Department of Endocrinology and Diabetes, Royal Perth Hospital, Perth, Western Australia Australia; 91 Department of Obstetrics and Gynecology, Tampere University Hospital, The Wellbeing Services County of Pirkanmaa, Tampere, Finland; 92 Medical and Clinical Genetics, University of Helsinki and Helsinki University Hospital, Helsinki, Finland; 93 Department of Paediatrics, University of Cambridge, Cambridge, UK; 94 Division of Endocrinology, Diabetes, and Metabolism, Cedars-Sinai Medical Center, Los Angeles, CA USA; 95 Big Data Institute, Li Ka Shing Centre for Health Information and Discovery, Nuffield Department of Medicine, University of Oxford, Oxford, UK; 96 Institute of Reproductive & Developmental Biology, Department of Metabolism, Digestion & Reproduction, Imperial College London, London, UK; 97 Division of Endocrinology, Metabolism and Diabetes, University of Utah, Salt Lake City, UT USA; 98 Center for Health Data Science, Faculty of Medical and Health Sciences, University of Copenhagen, Copenhagen, Denmark; 99 23andMe Research Institute, Palo Alto, CA USA

**Keywords:** Genetics research, Genome-wide association studies, Reproductive disorders

## Abstract

Polycystic ovary syndrome (PCOS) and its underlying features remain poorly understood. In this genetic study (*n* = 544,513), we expand the number of genetic loci from 16 to 29, and additionally identify 31 associated plasma proteins. Many risk-increasing loci were associated with later age at menopause, underscoring the reproductive longevity related to an increased oocyte number and/or availability across the lifespan. Hormonal regulation in the etiology of this condition, through metabolic and reproductive features, was emphasized. The proteomic analysis highlighted metabolic biology known to be related to PCOS. A polygenic risk score (PRS) was associated with adverse cardiometabolic outcomes, with differing relevance of testosterone and body mass index in women and men. Finally, while oligo-anovulation and anovulatory infertility are features of PCOS, we observed no impact of PCOS susceptibility on childlessness. We suggest that PCOS susceptibility confers balanced pleiotropic influences on fertility in women, and life-long adverse metabolic consequences in both sexes.

## Abstract (web summary)

Genome-wide analyses identify genetic loci and plasma proteins associated with polycystic ovary syndrome (PCOS). This study highlights the hormonal and metabolic foundations of the disease and explores the impact of polygenic risk for PCOS in both sexes.

## Main

Polycystic ovary syndrome (PCOS) is the most common reproductive endocrinopathy^1^, with impacts across the lifespan. The diagnostic criteria require two of three features—hyperandrogenism (HA), oligo-anovulation and/or polycystic ovarian morphology (PCOM)^2^. PCOS is the most common cause of anovulatory infertility and is associated with insulin resistance, conferring an increased risk of metabolic outcomes such as type 2 diabetes (T2D)^3,4^. Previous large-scale genetic studies demonstrated that PCOS is a complex polygenic disorder encompassing interactions among brain, metabolic and gonadal function^5^. High body mass index (BMI) and fasting insulin levels were identified as causal risk factors for PCOS^5^. Association at the *FSHB* locus highlighted the pituitary as a driver for PCOS^5,6^. The genetic susceptibility for later age at menopause was identified as causal for PCOS, linking PCOS etiology to the DNA damage response^6,7^. However, the small number of identified loci has limited further exploration^8^. There are no adequately powered prospective studies of women with PCOS beyond their reproductive years. Therefore, long-term health outcomes remain unknown. In addition, understanding of the genetic risk factors for PCOS on other health outcomes in women and men is incomplete.

To address these limitations, we conducted a meta-analysis in genome-wide association study (GWAS), including data from 20,818 cases and 523,695 controls, which doubled the number of women with PCOS compared to previous GWAS^6^ (Supplementary Table 1). We assessed the identified signals with a range of phenotypes encompassing three relevant mechanisms—metabolic pathways, hormonal regulation (including the hypothalamic–pituitary–gonadal axis) and the oocyte/follicle complement. We also conducted a complementary proteomic-based analysis to further identify the biology of this condition.

Previous studies used genetic instruments to explore the causal links from a range of phenotypes to PCOS, but not the downstream impacts of PCOS^9^. Many of these are likely to be a feature of ‘common soil’ effects, where several conditions stem from the same source; in this case, the adverse metabolic or hormonal background may be common to both women and men. However, there may be conditions where PCOS has a specific, additional, adverse effect. In particular, previous work implicated shared genetic influences on male-pattern balding and PCOS^5^, but whether PCOS risk variants impact disease risk for cardiometabolic and other health conditions in men has only been addressed to a limited extent^10^. Here the impact of PCOS on cardiometabolic disease, disorders of reproductive organs and mental health is examined in men and women.

## Results

### Genome-wide discovery for PCOS signals

**Fig. 1:**
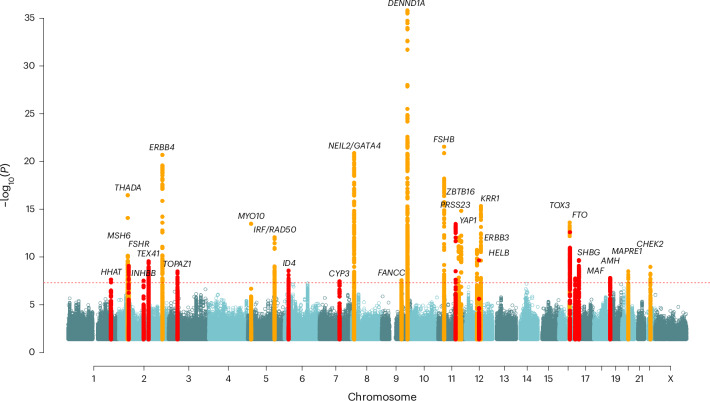
Manhattan plot showing the 29 genomic loci associated with PCOS. Two-tailed *P* values were generated by meta-analysis using an inverse variance weighted approach. Variants within 300 kb on either side of a genome-wide significant signal identified in this study are highlighted in red, those previously identified are in yellow. The dotted line indicates the genome-wide significance level of *P* = 5 × 10^−8^. Gene names indicate the consensus PCOS gene at each locus.

We identified 29 independent loci associated with PCOS (*P* < 5 × 10^−8^) in the all-ancestries meta-analysis, of which 13 had not previously been reported^5,6,11–13^ (Fig. 1 and Supplementary Table 2). The majority of the cohort is from European ancestry (93%), while the rest have African American (5%), East Asian (1%) or Hispanic ancestry (1%; Supplementary Fig. 1). These GWAS signals include a variant at *FTO* (rs8047587), confirming earlier findings^14^, and confirms the reported effect of increasing BMI on risk of PCOS^6,15^. Other PCOS signals have relevance to reproductive hormone pathways—*AMH* (rs732310), *INHBB* (rs6712151) and *SHBG* (rs1641518). Alongside the known European signal at *FSHB* (rs11031005), we report a signal at *FSHR* (rs13004711), replicating the association previously observed in Han Chinese women^11^. Three of the 29 loci (*INHBB*, *NEIL2*, *DENND1A*) had evidence of secondary signals (within 500 kb of the lead signal; Supplementary Table 3).

We also performed a BMI-adjusted model in a subset of the cohorts (Supplementary Table 4 and Supplementary Fig. 2). In this analysis, only the *FTO* locus was substantially attenuated (*P* = 0.019 after adjustment; Supplementary Fig. 3). To explore whether our findings were affected by differences in the criteria used to diagnose PCOS, we stratified studies based on the case definition. There were no differences in the effect sizes of the 29 PCOS signals by case definition (Supplementary Fig. 4), and they were comparable across individual studies (Supplementary Fig. 5). We also assessed the ten PCOS signals previously reported in East Asian women^11^ (Supplementary Table 5). Of these, all but three (variants near *C9orf3*, *INSR* and *SUMO1P1*) had statistically significant associations in our study (*P* < 0.005; for more details, see Supplementary Note). Finally, fine-mapping of the identified loci was performed, resolving the credible window for the associations (Supplementary Tables 6 and 7 and Supplementary Note).

### Identifying genes of interest

We used two approaches to identify PCOS risk genes, which we call ‘consensus genes’. First, we performed a literature review of all genes within 500 kb of the signals, prioritizing those with a reported link to one of the following four preselected processes: (1) reproductive function, (2) steroid metabolism and sex-hormone levels, (3) metabolic syndrome and (4) DNA damage repair. Evidence for genes linked to at least one of these processes is described in detail in Supplementary Table 8. Second, we used the GWAS-to-gene bioinformatic approach that leverages data on expression quantitative trait loci (eQTLs), protein QTLs (pQTLs), predicted deleterious variants and variant-based scoring methods to rank genes based on their causal likelihood (Supplementary Table 9)^16^. Findings from these two approaches were then harmonized for each PCOS signal (Supplementary Table 8).

**Fig. 2:**
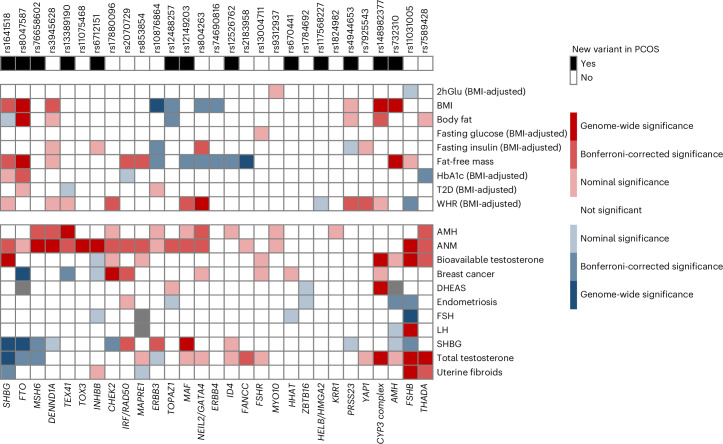
Heatmap of GWAS associations for the 29 PCOS loci with other relevant traits. Direction and the strength of association between the 29 PCOS risk-increasing alleles (top) with 20 other relevant traits and diseases with available GWAS summary statistics (right). Color coding indicates strength and direction (*z* scores) of associations—positive (red) and negative (blue). In the upper row of the heatmap, new PCOS loci identified in this study (black boxes) and previously reported loci (white boxes) are shown with corresponding lead variants. The top and bottom of the heatmap show metabolic and reproductive phenotypes, respectively. Gray boxes indicate missing variant-trait association data. Genes are presented in the lower *x* axis as ‘consensus gene’. DHEAS, dehydroepiandrosterone sulfate.

In 16 cases, both approaches prioritized the same gene. In the other cases, there was a strong rationale for prioritizing the literature-based gene instead of the bioinformatics-identified gene. For example, the gene *SHBG* was prioritized over *ATP1B2* at rs1641518, because the variant was also associated with circulating sex-hormone binding globulin (SHBG) levels (Fig. 2). The rs732310 variant was assigned to the *AMH* gene, based on the known functions of anti-Mullerian hormone (AMH) in inhibiting recruitment of ovarian follicles from the primordial follicle pool, inhibiting follicle-stimulating hormone (FSH) sensitivity of growing follicles and regulating gonadotropin-releasing hormone (GnRH)-dependent luteinizing hormone (LH) pulsatility^17,18^. Although rs732310 shows no association with AMH levels (Fig. 2), the GWAS data used for the AMH analysis were derived from normo-ovulatory women^19^, and may not reflect variations in AMH levels in women with PCOS, in whom the expression pattern of AMH differs^20^.

### Relationship of the identified loci with other phenotypes

Several of the 29 PCOS variants had previously been associated in GWASs for age at menopause (14 variants), age at menarche (6), female testosterone levels (7), BMI (8) and male-pattern baldness (2; Supplementary Table 10). We annotated our signals using publicly available GWAS results, focusing on the relationships between these variants and a range of metabolic, reproductive and hormonal phenotypes. All 29 PCOS signals showed at least nominal significance with one or more metabolic, reproductive and/or hormonal trait(s), providing evidence to support the multifactorial etiology and comorbidities (Fig. 2 and Supplementary Tables 11 and 12).

In validation of the substantial overlap between signals for PCOS and age at natural menopause (ANM), eight signals showed evidence of colocalization (posterior probability ≥ 0.75). PCOS signals at *FSHB*, *DENND1A*, *TOX3*, *RAD50* and *MAF/MAFTRR* were associated with ANM (Supplementary Table 13); conversely, the reported ANM signals at *FSHB, DENND1A, CASC22*, *BMP4*, *PPARG* and *MAF/MAFTRR* were associated with PCOS (Supplementary Table 14). At all eight colocalized signals, the PCOS risk-increasing allele conferred later ANM. The *FSHB* signal has been well described to affect other reproductive phenotypes, including age at menarche^21^ and dizygous twinning^22^. Other shared loci are also related to breast cancer pathways—*TOX3*, *CASC22* and *RAD50*^7^. Interestingly, the shared *PPARG* locus suggests an effect of metabolic pathways independent of BMI-related pathways.

A number of PCOS-associated loci also showed strong effects on hormone levels, particularly SHBG levels (including rs1641518 near *SHBG*). Approximately 30% (8/29) PCOS risk-increasing alleles had nominal association with lower SHBG levels. There were two loci in which PCOS risk alleles were associated with lower SHBG and higher total testosterone, suggesting a relationship driven by higher androgens; whereas three other loci, including *SHBG* and *FTO*, had lower SHBG and lower total testosterone, suggesting an SHBG-mediated effect (Fig. 2). Three of the signals had evidence of colocalization with SHBG levels, including the *CYP3* complex, *FTO* and *FSHB* (Supplementary Table 15). The signal at *FTO* is likely due to the established links between increasing BMI and decreasing SHBG^23^. The *CYP3* complex metabolizes oestradiol and testosterone^24^, with mouse knockouts showing substantially increased free testosterone levels^25^. The *FSHB* locus is associated with increased LH, which stimulates androstenedione, and, therefore, testosterone production^26^, which would lower SHBG levels.

There was additional evidence for the role of PCOS-associated loci impacting the regulation of gonadotropins and the functional ovarian reserve, that is, the pool of recruitable oocytes/follicles across a lifespan. The PCOS risk-increasing allele at the *FSHB* locus was associated with lower levels of FSH, and *INHBB* was nominally associated with lower FSH. Although the *FSHR* variant was not associated with FSH levels in 2,913 Europeans, the rs2268361-T variant found in Han Chinese women, which shows some evidence of linkage disequilibrium (LD; *R*^2^ = 0.3 in Europeans) with the variant reported here, was associated with lower FSH levels^27,28^ (Fig. 2 and Supplementary Table 11)*. FSHR* has also been linked to twinning rates and FSH levels using gene-based approaches^22^. At five loci, PCOS risk alleles were associated with higher AMH levels (*P* < 0.0017). All five loci overlap with the loci related to ANM and are associated with higher AMH. AMH is measured clinically to indicate the number of growing follicles, as a proxy of the functional ovarian reserve, and its concentrations are strongly related to age at menopause^18^. This suggests their involvement in the establishment and preservation of the functional ovarian reserve as a fundamental element of PCOS.

### Protein-based analysis

**Fig. 3:**
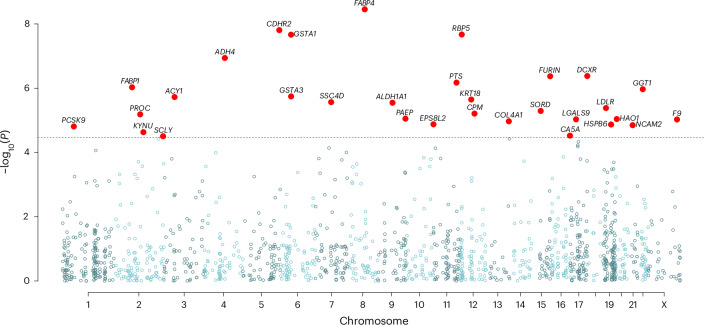
Manhattan plot showing the genomic positions of the genes for the 31 plasma proteins associated with ovarian dysfunction. Ovarian dysfunction was defined as the ICD-10 code E28, the supracategory that includes PCOS. Gene names and locations for proteins reaching significance are shown at the sentinel red dots. *P* values are two-tailed and come from linear regression analyses. The dotted line indicates a Bonferroni-corrected level of significance of *P* = 3.4 × 10^−5^. Significant proteins are (gene—protein) as follows: *ACY1*—aminoacylase 1; *ADH4*—alcohol dehydrogenase 4 (class II), pi polypeptide; *ALDH1A1*—aldehyde dehydrogenase 1 family member A1; *CA5A*—carbonic anhydrase 5A; *CDHR2*—cadherin-related family member 2; *COL4A1*—collagen type IV α 1 chain; *CPM*—carboxypeptidase M; *DCXR*—dicarbonyl and L-xylulose reductase; *EPS8L2*—EPS8 signaling adaptor L2; *F9*—coagulation factor IX; *FABP1*—fatty acid-binding protein 1; *FABP4*—fatty acid-binding protein 4; *FURIN*—furin, paired basic amino acid cleaving enzyme; *GGT1*—γ-glutamyltransferase 1; *GSTA1*—glutathione S-transferase α 1; *GSTA3*—glutathione S-transferase α 3; *HAO1*—hydroxyacid oxidase 1; *HSPB6*—heat shock protein family B (small) member 6; *KRT18*—keratin 18; *KYNU*—kynureninase; *LDLR*—low-density lipoprotein receptor; *LGALS9*—galectin 9; *NCAM2*—neural cell adhesion molecule 2; *PAEP*—progestagen associated endometrial protein; *PCSK9*—proprotein convertase subtilisin/kexin type 9; *PROC*—protein C, inactivator of coagulation factors Va and VIIIa; *PTS*—6-pyruvoyltetrahydropterin synthase; *RBP5*—retinol binding protein 5; *SCLY*—selenocysteine lyase; *SORD*—sorbitol dehydrogenase; *SSC4D*—scavenger receptor cysteine rich family member with four domains.

The levels of 31 plasma proteins were phenotypically associated with the International Classification of Diseases (ICD) 10 code E28, ovarian dysfunction, which includes PCOS (all *P* < 3.4 × 10^−5^; Fig. 3 and Supplementary Table 16). These included recognized metabolic disease-associated proteins such as PCSK9, LDLR, FURIN, FABP1 and FABP4. Other associated proteins metabolize hydroxysteroids, retinol and lipids, including ALDH1A1 and ADH4, that may regulate the metabolic response to a high-fat diet^29,30^. Other proteins are potential contributors to diabetes and metabolic disease, such as GGT1 (ref. ^31^), or their complications, SORD^32^. Finally, there were enzymes important for the biosynthesis of progesterone or testosterone, GSTA1 and GSTA3^33^, and fertilization and implantation, PAEP^34^. We used GProfiler to perform a combined pathway-based analysis using proteins drawn from either the protein-based approach or the GWAS-associated loci (Supplementary Fig. 6 and Supplementary Tables 17 and 18)^35^. The two discovery methods highlighted different biology, with only one pathway, ‘benzaldehyde dehydrogenase activity’, driven by both proteomic and GWAS findings (which included *FANCC*, a known DNA damage repair gene). The results show evidence of enrichment for pathways representing androgen binding, ovarian follicle development, neuregulin receptor activity and the PCSK9-LDLR complex consistent with the lipid abnormalities of PCOS^36,37^.

To build potentially causal pathways, we identified plasma proteins whose levels were associated with each of our PCOS GWAS variants, resulting in 299 proteins (*P* < 3.4 × 10^−5^; Supplementary Table 19). The PCOS signals at *ERBB3, ERBB4* and *ZBTB16* were associated with plasma levels of their encoded proteins, providing support for these consensus genes. The PCOS signal at *SHBG* was associated with levels of TNSF12 and TNSF13, encoded by genes in the same region, and related to apoptosis and regulation of steroidogenesis^38^. One PCOS signal at *RAD50/IRF1* accounted for 199 protein associations. This signal lies in a region on chromosome 2 that contains many immune-related genes—*IRF1*, *IL4*, *IL5* and *IL13*—and likely has a widespread impact on the plasma proteome. Another signal, overlapping the established obesity signal at *NEIL2*/*GATA4*, was associated with 29 plasma proteins. Other associated proteins included leptin, which is higher in obesity; PPY, a regulator of food intake; and several fatty acid-binding proteins, which regulate fatty acid uptake in adipose cells^39^. We resolved these to nine variant–protein pairs, and compared the variance explained in PCOS and in the respective protein levels (Supplementary Note and Supplementary Table 20), and for proteins linked to the signal at *FTO* BMI, similar to the logic of Steiger filtering^40^. All five *FTO*-associated proteins, NCAM2 associated with *ERBB3* and IGFBP2 associated with *MAF* appeared to be upstream (that is, more likely to be determinants, or common soil) of PCOS. The two other proteins associated with *MAF*, *CDHR2* and *CPM* may have their levels altered as a consequence of PCOS (Supplementary Fig. 7).

### Inferring causal impacts of PCOS on other comorbidities

The impact of PCOS on fertility, metabolic disease and mental health is well known, but few studies have used a genetic approach to uncover additional comorbidities^41^. Previous genetic causal modeling, using Mendelian randomization (MR) approaches, has shown that aspects of metabolic syndrome traits are risk factors for PCOS^6^. However, those MR studies did not determine whether PCOS had an effect on broader health status. Therefore, we calculated a PRS for PCOS comprising ~1.1 million genetic variants to explore the likely causal effect of PCOS on a number of other outcomes.

To identify phenotypes that may share genetic influences with PCOS and to determine potential differences between women and men, we performed PRS-based analyses in the UK Biobank study (independent of the discovery dataset). We used the PRS-CS software, a Bayesian regression framework that weights the effect size of each variant by the strength of its association (*P* value) in the GWAS meta-analysis^42^ to calculate a PRS. This was standardized; effect sizes (*β* or odds ratio (OR)) in Table 1 are reported per 1 s.d. increase in the PRS. To validate the score, we confirmed that it was associated with PCOS in women in the UK Biobank (*P* = 9 × 10^−27^) and that the odds of PCOS increased across increasing quintiles of the PRS (Supplementary Fig. 8). As expected, there was also a strong association between a higher PCOS PRS and an increased BMI in both women and men (Table 1).

**Table 1:** PCOS PRS associations with traits and diseases of interest

Trait	Women or men	*n*_total_ or *n*_cases/controls_	Age-adjusted PRS		BMI-adjusted PRS		Heterogeneity	
			*β* (s.e.) or OR (95% CI)	*P*	*β* (s.e.) or OR (95% CI)	*P*	*I*^2^	*P* for heterogeneity between sexes for age-adjusted PRS
Cardiometabolic								
BMI	Women	206,214	0.296 (0.012)	4 × 10^−143^	0.037 (0.012)	0.0017	91%	0.0007
	Men	175,708	0.243 (0.010)	1 × 10^−121^	0.027 (0.010)	0.009		
WHR (adjusted BMI)	Women	206,444	0.002 (0.0001)	2 × 10^−42^	0.002 (0.0001)	1 × 10^−37^	99%	<0.0001
	Men	176,000	0.0008 (0.0001)	4 × 10^−11^	0.0007 (0.0001)	5 × 10^−8^		
Obesity	Women	47,124/159,733	1.12 (1.10, 1.13)	1 × 10^−89^	1.02 (1.01, 1.03)	3 × 10^−4^	0%	0.6
	Men	44,499/131,861	1.12 (1.11, 1.13)	1 × 10^−87^	1.02 (1.01, 1.03)	3 × 10^−4^		
T2D	Women	6,713/117,099	1.13 (1.10, 1.16)	9 × 10^−21^	1.06 (1.03, 1.09)	2 × 10^−5^	17%	0.3
	Men	11,730/97,146	1.11 (1.09, 1.14)	2 × 10^−24^	1.05 (1.03, 1.07)	5 × 10^−6^		
CAD	Women	4,716/202,141	1.09 (1.05, 1.12)	7 × 10^−8^	1.06 (1.03, 1.10)	5 × 10^−5^	82%	0.02
	Men	15,857/160,503	1.04 (1.02, 1.06)	2 × 10^−6^	1.01 (0.99, 1.03)	0.3		
HbA1c (mmol mol^−1^)	Women	197,172	0.123 (0.013)	1 × 10^−21^	0.069 (0.013)	1 × 10^−7^	88%	0.004
	Men	168,088	0.187 (0.018)	2 × 10^−25^	0.076 (0.018)	3 × 10^−5^		
HDL (mmol l^−1^)	Women	179,166	−0.013 (0.001)	2 × 10^−45^	−0.005 (0.001)	4 × 10^−8^	92%	0.0004
	Men	155,355	−0.008 (0.001)	2 × 10^−23^	−0.002 (0.001)	0.01		
Triglycerides (mmol l^−1^)	Women	197,011	0.025 (0.002)	1 × 10^−38^	0.016 (0.002)	2 × 10^−16^	19%	0.3
	Men	168,122	0.029 (0.003)	2 × 10^−24^	0.019 (0.003)	2 × 10^−11^		
Hormonal/reproductive								
PCOS	Women	1,003/205,849	1.42 (1.33, 1.52)	9 × 10^−27^	1.27 (1.19, 1.36)	2 × 10^−13^	–	–
SHBG (nmol l^−1^)	Women	177,167	−1.593 (0.075)	6 × 10^−99^	−1.102 (0.076)	2 × 10^−47^	99%	<0.0001
	Men	154,245	−0.747 (0.042)	3 × 10^−69^	−0.614 (0.043)	8 × 10^−47^		
Testosterone (nmol l^−1^)	Women	164,553	0.014 (0.002)	4 × 10^−19^	0.012 (0.002)	5 × 10^−14^	100%	<0.0001
	Men	166,673	−0.144 (0.009)	2 × 10^−53^	−0.097 (0.009)	5 × 10^−25^		
FAI	Women	148,849	0.097 (0.005)	4 × 10^−71^	0.068 (0.006)	2 × 10^−34^	97%	<0.0001
	Men	153,352	0.296 (0.036)	1 × 10^−16^	0.297 (0.036)	1 × 10^−16^		
Breast cancer	Women	11,035/195,822	1.03 (1.01, 1.05)	0.008	1.03 (1.01, 1.05)	0.006	–	–
Age at menopause	Women	118,309	0.167 (0.015)	5 × 10^−30^	0.157 (0.015)	5 × 10^−26^	–	–
Childlessness^a^	Women	39,248/167,346	0.98 (0.97, 1.00)	0.009	0.99 (0.98, 1.00)	0.04	0%	0.98
	Men	37,567/137,139	0.98 (0.97, 1.00)	0.01	0.99 (0.98, 1.01)	0.4		
Other								
Depression^a^	Women	15,711/32,761	1.04 (1.02, 1.07)	1 × 10^−5^	1.03 (1.01, 1.05)	0.002	0%	0.5
	Men	8,802/34,069	1.03 (1.01, 1.06)	0.008	1.02, (1.00, 1.05)	0.1		
Asthma^a,^^b^	Women	29,117/135,476	1.02 (1.01, 1.03)	0.003	1.00 (0.99, 1.01)	0.9	33%	0.2
	Men	21,684/123,136	1.01 (0.99, 1.02)	0.3	0.99 (0.98, 1.01)	0.3		

^a^Controlled for Townsend deprivation index in addition to standard covariates.

^b^Controlled for smoking status (yes/no) in addition to standard covariates.

We calculated the PCOS PRS in women and men in the UK Biobank. Then, we tested for association between PCOS PRS and phenotypes of interest. We applied Bonferroni correction for multiple testing for associations with 17 phenotypes; hence, associations with *P* < 0.003 are considered statistically significant. Statistically significant heterogeneity between the sexes was considered when *I*^2^ > 80% and Cochran’s Q *P* value for heterogeneity < 0.004 (0.05/14 phenotypes analyzed).

HbA1c, hemoglobin A1c; HDL, high-density lipoprotein.

Because increased BMI is a risk factor for both PCOS and many of the expected metabolic comorbidities, we assessed the BMI-independent causal relationship between PCOS and metabolic outcomes in the following two additional analyses: (1) we included measured BMI as a covariate and (2) we generated and tested a second BMI-adjusted PRS. The association of the BMI-adjusted PRS with baseline BMI in the UK Biobank was substantially attenuated in women and men (Table 1 and Supplementary Table 21). The PRS analyses were replicated using an additional PRS tool LDpred2, which provided consistent results. In both women and men, a higher PCOS PRS was associated with increased risk of coronary artery disease (CAD), T2D and obesity (Table 1). It was also associated with cardiometabolic risk factors—higher waist-to-hip (WHR) ratio adjusted for BMI (WHR_adjBMI_), higher HbA1c, higher triglycerides and lower high-density lipoprotein cholesterol. Regarding hormonal/reproductive risk factors, a higher PRS was associated with lower SHBG levels and higher free androgen index (FAI) in both sexes (Table 1). These associations were also seen in the BMI-adjusted model analyses.

We tested for the presence of sex differences in the effects of the PRS on (1) cardiometabolic outcomes, where different effects of sex were observed for BMI, WHR_adjBMI_, HbA1c and high-density lipoprotein cholesterol; and (2) hormonal/reproductive outcomes, where differential sex-related effects were observed for SHBG, total testosterone and FAI (Table 1). There has been increasing interest in associations between reproductive diseases and mental health^43,44^, but we found only a nominal association between the PRS and depression in women after BMI adjustment.

We used MR to provide additional evidence of causality (Supplementary Tables 22 and 23). While MR provides more robust causal inference, there are caveats—outcome data are often in men and women combined, and with only 29 variants, this analysis is likely underpowered. Most of the associations with nonreproductive phenotypes were not significant, supporting the idea of an underpowered analysis. Interestingly, the MR analysis showed associations with two noncardiometabolic outcomes—depression and asthma—with no attenuation when controlling for BMI.

### Pleiotropic effects of PCOS on reproductive outcome

Consistent with the overlap across signals for PCOS and ANM, PCOS susceptibility was associated with later ANM in both PRS (Table 1) and MR analyses (Supplementary Table 23). We also observed an apparent effect of susceptibility to later ANM on higher PCOS risk (Supplementary Table 23), indicating a bidirectional relationship. Given the importance of DNA repair as a mechanism that regulates ANM, we tested whether the same pathways also contributed to PCOS. We performed MR analyses for PCOS with reported variants for ANM stratified by a previous annotation of a DNA-repair pathway^7^. Both strata of menopause variants indicated an effect of later ANM on higher risk of PCOS (Supplementary Table 23). However, the estimated effect of ANM was larger using non-DNA-repair ANM variants than when using DNA-repair variants (for the difference between estimates, *P* = 1.5 × 10^−6^; Supplementary Fig. 9), suggesting a role for sex-hormone-related pathways common to PCOS and ANM. This is highlighted by the shared signal at *FSHB* associated with lower FSH levels, higher PCOS risk and later age at menopause.

The subset of DNA-repair-related ANM variants that are associated with PCOS has selective effects. For example, ANM variants at *BRCA1* and *BRCA2*, genes involved in homologous recombination repair of double-strand DNA breaks, are robustly associated with an earlier ANM but not with PCOS (*P* = 0.94 and *P* = 0.11, respectively). Conversely, *CHEK2*, known to maintain DNA integrity through checkpoint control^45^, is associated with a later ANM, greater risk of PCOS and higher serum AMH levels in PCOS^7,18^.

Given that PCOS is one of the most common conditions resulting in reduced fertility, the PCOS PRS showed unexpected nominal associations with lower risk of childlessness, although not confirmed in the MR analysis. We therefore examined the impact of PCOS susceptibility on eight infertility phenotypes in the Copenhagen Hospital Biobank (CHB)^46^, applying Bonferroni correction for multiple testing (0.05/8 phenotypes analyzed = *P* < 0.006; Supplementary Table 24). A higher PCOS PRS was associated with increased risk of infertility in women (OR = 1.03, *P* = 0.02 after age adjustment; OR = 1.04, *P* = 0.00011 after age and BMI adjustment). The stronger association after adjustment for BMI suggests that, in addition to affecting fertility through BMI, PCOS also affects fertility through BMI-independent mechanisms, such as HA.

Interestingly, the PCOS PRS was associated with an increased number of oocytes aspirated during in vitro fertilization treatment (*β* = 0.025, *P* = 1 × 10^−4^ after age adjustment; *β* = 0.027, *P* = 2 × 10^−5^ after age and BMI adjustment). In a separate dataset of 812 women, two of our loci, *ZBTB16* and *SHBG*, were associated with larger ovarian volume, which correlates with oocyte/follicle number and is a symptomatic presentation of PCOS^47^. These findings suggest a greater available oocyte pool in PCOS.

There was also support for the hypothesis that some genetic PCOS susceptibility might exhibit a balancing pleiotropy effect on reproductive success. We assessed the links from PCOS to age at first birth^48^, age at last birth^48^, childlessness^49^ and number of children^49^ using publicly available GWAS datasets (Supplementary Table 23). There were no apparent associations with childlessness or number of children. The latter result was confirmed in the CHB data, in which there was no association between the PCOS PRS and completed family size (*P* = 0.07) or rates of pregnancy (*P* = 0.25; Supplementary Table 24). However, there was a nominally significant association with later age at last birth when data were controlled for age and BMI (*P* = 0.01). We further assessed the association between PCOS and age at last live birth in the UK Biobank in 1,003 women with PCOS and 205,849 controls. PCOS was associated with later age at last birth (*β* = 0.46 years, *P* = 0.04 after age adjustment; *β* = 0.81 years, *P* = 0.0003 after age and BMI adjustment). These results could be explained by a longer reproductive window or by a shifting of the window, and the lack of association with childlessness indicates that some compensatory mechanisms exist.

## Discussion

This study expands the number of PCOS genome-wide significant loci from 16 to 29. The locus at *FTO*, well established for obesity, highlights the link among PCOS, metabolic syndrome and obesity. Other signals at *SHBG*, *FSHR* (associated in a European GWAS) and the *CYP3* complex highlight hormonal regulation in the etiology of PCOS. Alongside these results, we also present proteins that are associated with ovarian dysfunction. The protein associations confirm some candidate genes at GWAS loci (*ERBB3, ERBB4* and *ZBTB16*).

The identified loci underscore the sex hormonal origins of PCOS. While our literature-based method might be biased toward particular pathways, there was clear evidence of important biology. We identified signals at *FSHB*^50^ and *FSHR*^11^, highlighting the role of pituitary gonadotrophs (LH and FSH) in ovarian stimulation. Other consensus genes *SHBG*, *INHBB*, *AMH* and *TEX41*—this last robustly associated with AMH levels—also point to hormones related to ovarian folliculogenesis, and feedback on the hypothalamic–pituitary–gonadal axis. Inhibin B is secreted by granulosa cells of small to large antral follicles and inhibits FSH release, ensuring the growth of one dominant follicle^51,52^, and the *INHBB* variant was nominally, inversely associated with FSH levels. Circulating AMH levels reflects the number of growing small antral follicles and AMH reduces FSH sensitivity of growing follicles^18^. Furthermore, AMH inhibits aromatase activity at the level of a growing follicle and increases LH-dependent GnRH pulsatility at the hypothalamus^53^. Finally, SHBG is a binding protein for androgens, thereby regulating free and bioavailable androgen levels^54^. In men, there is a corresponding increase in FAI that explains the connection between PCOS variants and male-pattern balding^6^. In summary, PCOS risk is affected by a number of classical and well-established sex hormonal pathways.

Many of the identified PCOS loci overlap with those associated with age at menopause with PCOS risk alleles conferring later ANM. Two possible mechanisms could explain, perhaps in tandem, the links between these two phenotypes. First, several overlapping variants are related to genes linked to DNA-repair mechanisms such as *MSH6*, *CHEK2* and *RAD50*. The partitioned MR analysis suggested that both DNA damage repair and non-DNA damage repair (predominantly hormonal pathways) were causal for PCOS, but the latter had a stronger influence. While ANM is thought to be impacted by a range of pathways linked to DNA repair, the signals shared with PCOS might be related to more specific mechanisms, such as *CHEK2*, where the effect is to have DNA-damaged oocytes persist for longer^7^. Thus, there may be follicles with DNA-damaged oocytes that remain in the ovary because the DNA checkpoint removal mechanism failed. In PCOS, this may reduce oocyte atresia, leading to continuous AMH expression and thereby stronger inhibition of primordial follicle recruitment (associated with later ANM) and reduced FSH sensitivity (contributing to the polycystic ovary morphology seen in PCOS)^17,18^. In addition, PCOS was not associated with *BRCA1* or *BRCA2* variants, which appear to influence earlier ANM based on less functional DNA-repair mechanisms and potential loss of damaged oocytes^7^.

Second, changes in hormonal levels may increase follicle numbers as demonstrated for increased androgen levels^55^ and activin, the product of two inhibin βB subunits^56^. It is also possible that hormone levels reduce depletion of the primordial follicle pool, causing a later end to the reproductive window, as has been demonstrated for the variants causing lower FSH levels^57^. Moreover, the variant in *FSHB* was linked to less follicle selection across the reproductive lifespan, potentially leading to a greater follicle pool consistent with PCOS. Similarly, increased serum AMH levels, seen in PCOS patients, reduce the rate of primordial follicle recruitment and may thereby slow follicle pool depletion, leading to later menopause^17,18^. Observational studies suggest that women with PCOS or PCOS symptoms have children as often as asymptomatic women, with reproductive success in the long term^58–61^. In both our PCOS PRS and epidemiological analysis of women with PCOS in the UK Biobank, there was a suggestion of later age at last birth and this effect was also seen in the CHB data. This is consistent with a previous finding that a longer or shifted window of reproduction was required for the same cumulative family size in women with PCOS^61^. The main cause of infertility in PCOS is irregular ovulation. The relative infertility at younger ages may be balanced by improved ovulations at later ages^62,63^.

In summary, the new loci contain risk genes expected to increase the follicle complement in PCOS^64,65^. This finding supports the Rotterdam diagnostic criteria for PCOS, highlighting PCOM (number of growing small antral follicles), HA (hormone regulation), and related irregular ovulation and menses as primary etiologic features of PCOS^2^.

The score-based analyses stressed the link to metabolic diseases, with a number of strong associations between PCOS and clinical endpoints, mirroring observed associations^66^, and previous studies^10,40^. A higher PCOS PRS was associated with higher BMI in both women and men; thus, much of the effect on cardiometabolic diseases seen in the BMI-unadjusted models is through the ‘common soil’ impact of BMI. However, many of the associations remained significant in women (although substantially attenuated), and not in men, after controlling for BMI. These female-specific effects, unrelated to BMI, suggest a shared causal factor between PCOS and metabolic disease. A plausible mechanism is that higher androgen levels in PCOS are a risk factor for CAD^67–69^. Although testosterone and other androgens decrease to the same level as in controls after menopause, the continued lower SHBG in women with PCOS after menopause and the lasting impact of androgens during reproductive age on physiology may result in long-term increased CAD risk^70,71^.

The proteins associated with reproductive dysfunction stress the links between reproductive phenotypes and the metabolic syndrome. Associations were seen with classical adiposity and metabolic proteins, including leptin and furin. Other associated proteins are vital to cholesterol metabolism, such as PCSK9 and the low-density lipoprotein receptor, which are important for both cardiovascular risk and steroidogenesis. There were also proteins that contribute to the metabolic response to a high-fat diet. It is important to consider that most of the women in whom the protein-based analysis was done were assessed after the end of their reproductive window. The data again implicate lower SHBG and higher free androgen levels in PCOS after menopause^70^, and potentially sustained effects of HA even after the reproductive years. Thus, these results and the score-based analyses together suggest that there is an ongoing, adverse pattern of cardiometabolic health in women with a genetic risk for PCOS.

## Conclusions

Here we identify genetic regions and proteins associated with PCOS. The genomic loci appear to primarily implicate hormonal pathways as the causal factors for PCOS, while the proteins stress the common factors that influence PCOS and metabolic disease, particularly pathways related to increased BMI. Our findings highlight important links between PCOS and T2D and CAD, through mechanisms that are related to and also independent of adiposity. We also expand our understanding of the factors affecting the ovarian follicle complement on the condition, including both hormonal influences and specific DNA-repair mechanisms, and their role in PCOS. We also demonstrate some evidence of balanced pleiotropy conferred by PCOS genetic susceptibility that maintains the high prevalence of PCOS in the population.

## Methods

### Ethics

This study complies with all relevant ethical regulations—details of the specific ethical approval for the studies contributing to the meta-analysis, or other analyses can be found in the Supplementary Note. All identifiable participants in all cohorts provided written informed consent. Anonymized data were studied under a waiver of informed consent.

### Study characteristics

This study included summary statistics of our previous meta-analysis, including 3,981 cases and 17,322 controls of European ancestry after removing overlapping samples (Supplementary Table 1; described in detail in ref. ^6^). An additional 16 studies from 13 cohorts were included for the current GWAS meta-analysis, resulting in 20,818 cases and 523,695 controls (Supplementary Table 1). The newly added cases included all ancestries, of which 87% cases were from European origin, 10% from East-Asian origin and the remaining were either from Hispanic or African-American ancestry. Included women with PCOS were diagnosed using three different PCOS criteria, namely (1) the clinical diagnostic criteria (either the Rotterdam or the National Institutes of Health (NIH) criteria), (2) women diagnosed using the electronic health record (EHR) ICD-8, ICD-9 or ICD-10 codes, or (3) self-reported cases (see more details in Supplementary Note and Supplementary Table 25). The Rotterdam criteria diagnosis is based on the presence of at least two of the three criteria—ovulatory dysfunction, HA and PCOM. The NIH criteria only require the presence of ovulatory dysfunction and HA. Detailed description of each cohort can be found in Supplementary Table 1. In Supplementary Fig. 1, we have visualized the distribution of diagnostic criteria and ethnic groups of the newly added cases and previous meta-analysis results^6^ combined.

### Data collection and quality control

Summary results of GWAS using a case-control setting were provided by the studies contributing to the meta-analysis. At the study level, the analyses were adjusted for age, principal components and BMI (only for BMI-adjusted analyses). Central quality control was performed by two independent analysts using the EasyQC pipeline^72^. Variant exclusion filters used included—(1) minor allele frequency < 1%, (2) imputation quality (*R*^2^) < 0.3 or info of <0.4 for MACH and IMPUTE2, respectively^72^.

### Meta-analysis

A fixed-effect, inverse-weighted-variance meta-analysis approach was used with the collected summary statistics from the individual studies. Either GWAMA^73^ or METAL^74^ was used as a meta-analysis tool. We performed meta-analyses for all ancestries combined and only for European ancestry combined. These meta-analyses were carried out using two models—age-adjusted and age and BMI-adjusted—given the association between obesity and PCOS^5^. Variants present in at least three strata were reported and used in further analyses.

These meta-analysis results were then combined with the previously published genome-wide meta-analysis summary statistics^6^ to increase the statistical power and discover further associations with PCOS status. We called this analysis ‘the two-strata meta-analysis’. The resulting sample size was 456,570 (15,634 cases and 440,936 controls; Supplementary Table 1). As previous research had found no substantial heterogeneity in variant discovery as a function of different diagnostic criteria^6^, studies with any method of case ascertainment were combined. Variants present in all strata were reported and used in the follow-up analyses. Identified variants were annotated and investigated further with regard to their biological function using FUMA^75^. Forest plots for comparing the effect sizes across the strata in the meta-analysis were made using the ggplot2 package in R.

Furthermore, we compared the effect sizes across different phenotype definitions used; PCOS definitions based on EHRs, clinical diagnosis and self-reports were included in this comparison. EHRs were based on de ICD-8/ICD-9/ICD-10 codes for PCOS (E28.2/256.4/256.9, or Hirsutism L68.0/704.1/704 and Irregular Menses N91.X, N92.5, N92.6/626.X/626). Clinical diagnosis was based on either the NIH criteria or the Rotterdam criteria. Additional meta-analysis was performed to statistically test for heterogeneity across these three PCOS definitions. In addition to visually inspecting forest plots for the meta-analysis, Cochrane’s Q *P* value and *I*^2^ were used for assessing heterogeneity. A *P* value below 0.05 was considered statistically significant.

The summary statistics from the age-adjusted meta-analyses were further combined with the previously published summary statistics from 23andMe to increase the statistical power^6^. We called this analysis ‘the three-strata meta-analysis’. The resulting sample size was 544,513 (20,818 cases and 523,695 controls; Supplementary Table 1).

To assess the effects of the lead 29 GWAS variants (*P* < 5 × 10^−8^) in the BMI-adjusted model, we performed an individual variant look-up in the summary statistics of the BMI-adjusted model for the three-strata (Supplementary Table 2) and two-strata (Supplementary Table 4) analyses. For this single variant association study, associations were considered statistically significant if they passed the Bonferroni correction for 29 variants (*P* < 0.05/29 = 0.0017).

We assessed the lead GWAS variants (*P* < 5 × 10^−8^) by examining their relationship with 20 related metabolic, hormonal and reproductive phenotypes with available GWAS results data. Except for LH and FSH all other traits were publicly available (Supplementary Table 10). The heatmap was drawn using the ‘pheatmap’ library in R (v3.6.1). Finally, colocalization analysis was performed on the three-strata data using the R package ‘coloc’^76^, based on a window 500 kb either side of the sentinel signals.

### Fine-mapping

To identify a credible set of variants containing the most likely causal variant underlying our association signals, we conducted fine-mapping using the shotgun stochastic search method as performed in FINEMAP^77^. We used summary statistics from our two-stage summary GWAS meta-analysis results without the data from 23andMe, and considered all variants within 1 Mb± from our tag variants. We used two different contributing studies as LD references to perform fine-mapping. First, we used unrelated (up to second degree), European ancestry women from the MyCode Community Health Initiative Study (DiscovEHR; *n* = 47,061) with genetic data imputed to the 1000 Genomes Phase III global reference panel. European ancestry was inferred using genetic data as described elsewhere^78^. Second, we used an unrelated dataset of European-ancestry females (*n* = 36,890) in the EHR-linked biobank at Vanderbilt University Medical Center (BioVU). Genetic data were imputed to the Haplotype Reference Consortium and European ancestry was defined by principal components^79^. We assumed a single causal variant for all loci, and for four loci with evidence of a secondary signal, we also performed fine-mapping assuming two causal variants.

### Functional mapping and annotation of GWAS

Functional mapping and annotation of GWAS were performed with FUMA, and further annotation of the association results with PhenoScanner (date accessed on 25 March 2022)^80,81^. FUMA analyses were performed using the summary statistics for (1) the top 29 PCOS-associated variants in the three-strata meta-analysis and (2) the genome-wide two-strata meta-analysis. Unless specified otherwise, the default settings were used in the FUMA analyses for both SNP2GENE and GENE2FUNC^75^.

### Proteomic analysis

Proteomic analysis using logistic regression for the association of normalized plasma protein levels with disease was run in the ~22,000 women with data from the Olink panel of plasma proteins, aged 56.5 ± 8.1 years^82^. Here the outcome was the first occurrence data, and to maximize sample size, we used a diagnosis of any of the ICD-10 code E28, the supergroup that includes PCOS. Proteins were considered significantly associated if they passed a Bonferroni-corrected *P*-value threshold of 3.4 × 10^−5^. The generation of the protein data is described elsewhere^83^.

Separately, we performed a protein PheWAS for each variant identified in the GWAS meta-analysis, using the total sample of ~44,000 (men and women) and Olink data to identify proteins linked to our PCOS signals. Again, we used a Bonferroni-corrected *P*-value threshold of 3.4 × 10^−5^. Once this panel of proteins had been identified, we prioritized proteins significantly associated with the ICD-10 code E28 diagnosis, as defined above. Finally, once we had established our variant–protein pairs, we attempted to establish the position in the causal pathways by considering the relative *R*^2^, with those variants that had a higher *R*^2^ with PCOS suggesting that the protein was downstream of PCOS, and vice versa.

### Annotating genes of interest

First, we performed a literature review of all genes within 500 kb of the 29 signals to identify genes with a reported link to one of the following four processes: (1) reproductive function, (2) steroid metabolism and sex-hormone levels, (3) pathways related to metabolic syndrome and (4) DNA damage repair, selected due to reported links between PCOS and age at menopause for which DNA damage repair is the dominant process^7^. The literature search was conducted using PubMed and the Cochrane databases. For each gene, the most important findings regarding the four preselected processes are summarized in Supplementary Table 8.

Second, we used the established GWAS-to-genes pipeline, which integrates genomic and functional data through multiple gene annotation methods to highlight likely causal genes at each of the identified signals, as described elsewhere^16^. Briefly, tissue enrichment for GWAS associations was performed using LD score regression to identify key tissues for annotations with tissue-specific datasets. Then, a gene score is generated from the following panels of annotations: (1) the closest gene to the signal scored 1.5 points. (2) eQTL^84^ colocalization from both SMR-HEIDI and the ‘coloc’ package^76^ was scored 1.5 points, or 1 if only from one of these. An additional point was given to genes with eQTLs at secondary signals. (3) Colocalization with pQTL derived from plasma^82,85^ scored the same as for eQTLs. (4) Coding variants, with variants of deleterious or damaging predicted consequence in LD with GWAS PCOS signals, were scored 1.0 point, or only 0.5 points if the coding variants were predicted to be benign or tolerated. (5) Genes targeted by enhancers that overlapped with or were correlated with GWAS PCOS signals were scored 1.0 point. (6) Polygenic priority score prioritized genes at each locus were scored 1.5 points^86^.

### Gene-set enrichment analysis

To perform gene-set enrichment analysis that leveraged information across both the proteomics and the implicated genes, we used GProfiler, selecting either the consensus gene or the associated protein from the proteomics analysis. Clustering of the pathways was done using an index of dissimilarity based on the shared genes across the enriched intersections of each pathway^16^.

### PRS analyses in the UK Biobank

We used the PRS-CS software to calculate a PRS for PCOS, which is a Bayesian regression framework that applies continuous shrinkage parameters to estimate posterior effect sizes^42^. This work was performed in the UK Biobank, a population-based cohort of ~500,000 individuals in the United Kingdom^87^, which was independent of the discovery GWAS sample. The tuning or global shrinkage parameter phi = 1 × 10^−4^ that optimized the association of the PRS for PCOS in the UK Biobank, as previously reported, was used^10^. Using this method, our PCOS PRS included 1,119,009 genetic variants. In the same UK Biobank sample, we replicated these analyses using another PRS tool, LDpred2, that uses a Bayesian shrinkage model^88^.

To identify women with PCOS in the UK Biobank study, data from self-report, primary-care clinical events and/or ICD-9 and ICD-10, as previously reported,^10^ were used. We binned women with or without a diagnosis of PCOS by their quintile of PRS and used logistic regression to determine the odds of PCOS for each quintile using the lowest quintile as a reference. Women with a PCOS PRS in the highest quintile had an increased odds of PCOS (OR = 2.41, 95% CI = 1.96–2.98; *P* = 2 × 10^−16^). Thus, our PCOS PRS is able to represent the genetic risk for PCOS in women in the UK Biobank.

Ascertainment of cardiometabolic and androgenic phenotypes has been previously reported^10^. All other phenotypes, including measures of fertility and longevity, asthma and mental health disorders, were based on a composite of self-reported measures, diagnosis codes from hospitalization records and age at diagnosis (Supplementary Note). We used linear and logistic regressions to analyze the associations between continuous and dichotomous phenotypes and the PCOS PRS, respectively. We adjusted all analyses for age, age squared, genotyping array, the UK Biobank assessment center and the first ten genetic principal components; for asthma and psychological outcomes, we additionally controlled for the Townsend deprivation index, and, for asthma, we further controlled for smoking status. Adjustment for BMI was performed in the following two ways: (1) a measured BMI was included in the model as a covariate, and (2) we constructed a score based on the GWAS meta-analysis where the genetic associations were adjusted for BMI.

### PRS analysis in CHB based on the Danish Registries

PRSs for PCOS were calculated using LDpred2 (ref. ^89^). These genome-wide scores were calculated using the meta-analysis excluding data from 23andMe. Autosomal genotype data from 138,669 individuals in the CHB were filtered to only include variants present in a set of 1,054,330 reference variants recommended by LDpred2 developers. Missing genotype information was imputed to be the reference allele for the affected locus. GWAS summary statistics were preprocessed with MungeSumStats.

The completed family size was determined by counting the number of live births from the Medical Birth Registry^90^. This study was initiated in 1973, and data are considered complete. Only women born in the years 1957–1973 were included in this analysis, the youngest would be 45 years old, and 61 years old when data collection ended (31 December 2018). Data were treated as count data, and we tested to determine whether there was equidispersion, underdispersion or overdispersion using the AER R package (v.1.2.10). We found significant underdispersion (dispersion = 0.60, *P* < 2.2 × 10^−16^). Consequently, data were analyzed using a Conway–Maxwell–Poisson distribution.

From the Medical Birth Registry, we also identified the age at first birth and last birth (expressed in days). Data were analyzed using a linear regression, and model fit was inspected from residuals. There were no signs of deviation from a Gaussian error.

The Danish IVF registry was initiated in 1994 and contains data on all treatments and procedures related to medically assisted reproduction. Reporting is mandatory for both private and public clinics. Furthermore, there is information on any procedure-related treatments and their duration. Female infertility was defined using the 628 (ICD-8) and N97 (ICD-10; excluding N97.4) in the National Patient Registry (public hospitals only^91^) and ‘female cause’ in the IVF Register. Male infertility was defined using the 606 (ICD-8) and N46 (ICD-10) in the National Patient Registry and ‘male cause’ (excluding male infertility due to sterilization) in the IVF Register. For the number of oocytes, we extracted all aspirations performed between 18 January 1994 and 31 December 2018. The mandatory reported data were changed in 2005, and, thus, we analyzed the two time periods (1994–2005 and 2006–2018) separately and meta-analyzed. We additionally extracted information on treatment (Klomifen, HMG-FSH, GnRH-A, Estrogen, Progesterone, HCG) and the number of treatment days before the aspiration. Both time periods were overdispersed and were analyzed using a negative binomial distribution. To take into account multiple aspirations for a single woman, we included an individual random term.

Finally, we investigated the number of cycles before a woman got pregnant or ceased treatment. Each woman was only included until the first pregnancy. All transfer or insemination attempts were summarized, and analyzed using a Conway–Maxwell–Poisson distribution, as data were underdispersed. Furthermore, a term for zero inflation was also tested, as only 81% of the population became pregnant. A model that included a zero-inflation term was found to fit significantly better (*P* < 2.2 × 10^−16^, likelihood-ratio test). This was also substantiated by lower Akaike information criterion and Bayesian information criterion scores.

All models were fit using glmmTMB (/services/tools/R/4.0.0/R_PACKAGES.txt:glmmTMB ‘1.1.5’), and no rate models, except the number of cycles until pregnant, had indications of zero inflation.

GWAS catalog accessions for calculated bioavailable testosterone, total testosterone and SHBG are GCST90012102, GCST90012106 and GCST90012112, respectively.

### MR analysis

MR analysis was performed using two-sample inverse-weighted methods^92^. In addition, the intercept from the MR-EGGER^93^ was calculated to provide a test of directional pleiotropy and the I^2^ metric to assess general heterogeneity of the variants. Data for the outcomes were taken from the most recent genome-wide study for each outcome (thus, in most cases, these data were not sex-specific). To correct for any impact of the role of BMI on analyses multivariate inverse-weighted method^94^ was implemented. The βs for these variants to BMI were taken from the most recent GIANT consortium study that combined GWAS meta-analysis data with that from UK Biobank^95^. For the analysis of association between menopause variants and PCOS split by evidence for a DNA damage effect, the variants were classified based on the proximity to a known DNA damage repair gene as discussed in ref. ^7^.

### Reporting summary

Further information on research design is available in the Nature Portfolio Reporting Summary linked to this article.

## Online content

Any methods, additional references, Nature Portfolio reporting summaries, source data, extended data, supplementary information, acknowledgements, peer review information; details of author contributions and competing interests; and statements of data and code availability are available at 10.1038/s41588-026-02543-9.

## Supplementary information

Supplementary Information Supplementary Note, Supplementary Table 25 and Supplementary Figs. 1–9.

Reporting Summary

Supplementary Tables Supplementary Tables 1–24.

## Data Availability

Genome-wide data from the two-strata model (that is, the meta-analysis without the data from 23andMe) will be available from the GWAS catalog, accessions GCST90570579 and GCST90570580. Data from 23andMe can be made available to qualified investigators who enter into an agreement with 23andMe that protects participant confidentiality; details on the access procedures for 23andMe, including links to data access request forms and details of time frames for requests, can be found at https://research.23andme.com/dataset-access/.

## Code availability

No custom code was used in this project, details of the reference for code used can be found in the methods.
