# Supplementary material for: Genomic analyses implicate hormonal and metabolic dysregulation in polycystic ovary syndrome
Source: Nat Genet. 2026 Apr 23;58(5):1040–50. doi: 10.1038/s41588-026-02543-9 (PMC13175888; doi:10.1038/s41588-026-02543-9)
Supplement: Supplementary file 1 — Supplementary Note, Supplementary Table 25 and Supplementary Figs. 1–9. [file 41588_2026_2543_MOESM1_ESM.pdf]

nature genetics

Supplementary information https://doi.org/10.1038/s41588-026-02543-9

Genomic analyses implicate hormonal and metabolic dysregulation in polycystic ovary syndrome

In the format provided by the authors and unedited

Supplementary Information

Index

Supplementary Figures ... 2
- Supplementary Figure 1 ... 2
- Supplementary Figure 2 ... 3
- Supplementary Figure 3 ... 3
- Supplementary Figure 4 ... 4
- Supplementary Figure 5 ... 5
- Supplementary Figure 6 ... 10
- Supplementary Figure 7 ... 11
- Supplementary Figure 8 ... 11
- Supplementary Figure 9 ... 12

Supplementary Table ... 13
- Supplementary Table 25. International Classification of Disease (ICD) codes used to identify women with PCOS in electronic medical records ... 13

Supplementary Note ... 14
- Supplementary Methods ... 14
  - Subjects ... 14
  - Summary-data-based Mendelian Randomisation (SMR) Analysis ... 14
  - Polygenic Risk Score (PRS) Phenotype Definitions in the UK Biobank ... 15
- Supplementary Results ... 17
  - SMR analysis in PCOS-relevant tissues ... 17
  - Analysis of previously reported loci ... 17
  - Fine-mapping of PCOS signals ... 17
  - Protein analysis ... 18
- Additional Cohort-specific Details ... 19
  - FinnGen ... 19
  - Estonian BioBank ... 19
  - Dutch Cohort ... 20
  - Genes and Health ... 20
  - Danish Blood Donors Study (DBDS) Genomic Consortium ... 20
- Other Funding and Acknowledgements ... 21
- Address Changes ... 22
- Ethics statements ... 23
- Supplementary References ... 25

Supplementary Figures

Supplementary Figure 1

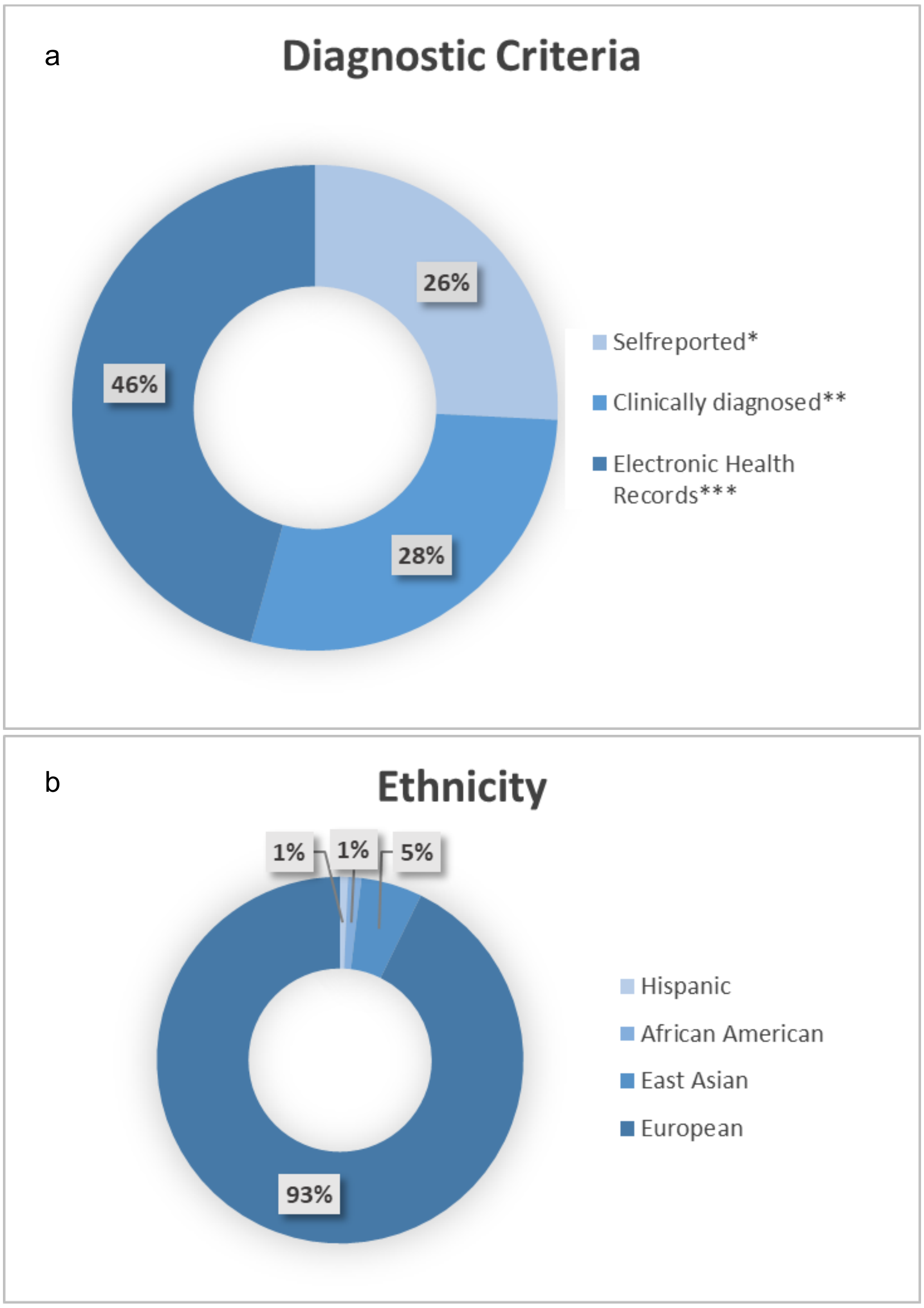

Distribution of the cohort by diagnostic criteria and ethnicity. a) identifies the source of subjects as self-reported, clinically diagnosed or from electronic health records. b) indicates the ethnicity of the included subjects.

Supplementary Figure 2

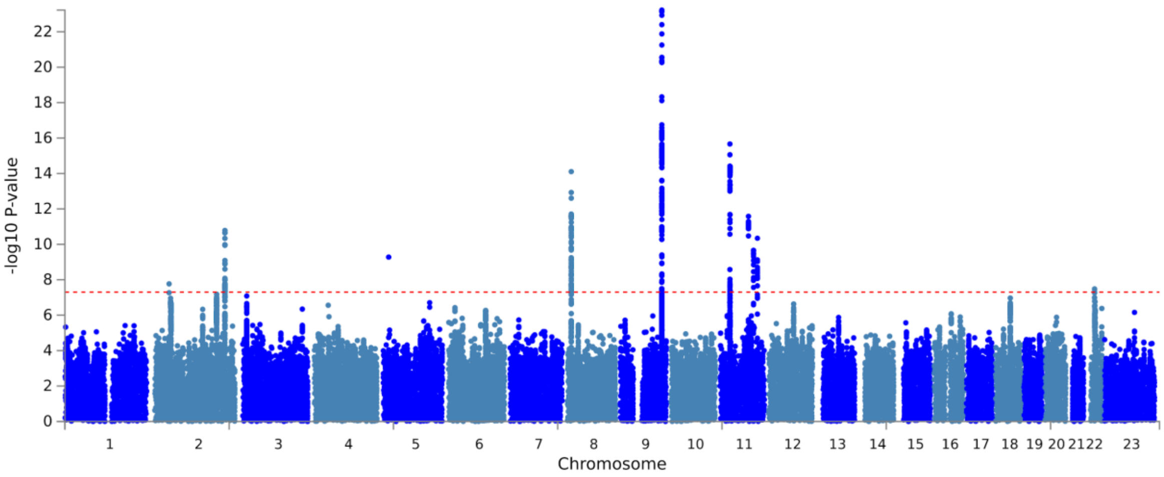

Manhattan plot of the genome-wide data adjusting for body mass index (BMI). The dotted red line indicates the genome-wide significance level of $p=5\times10^{-8}$, N= 295,814 samples.

Supplementary Figure 3

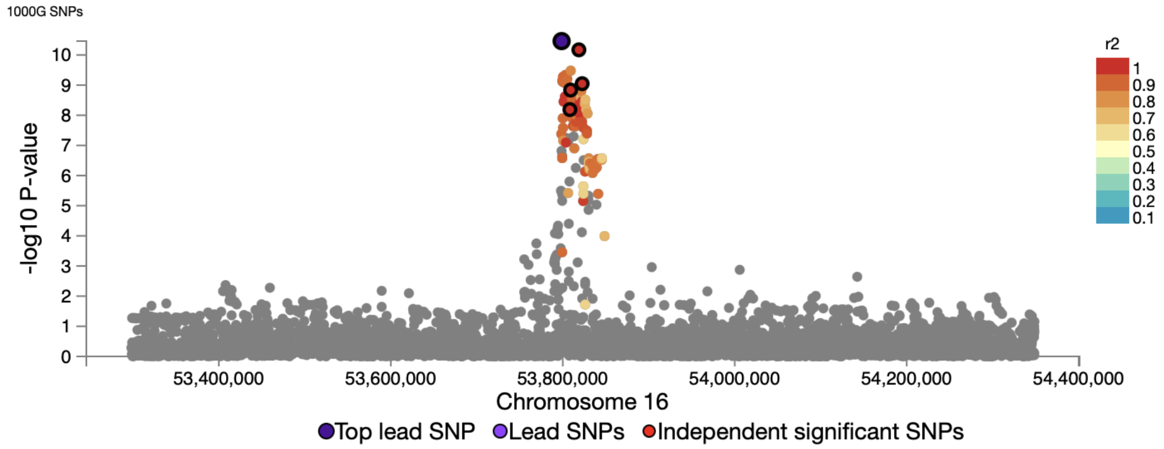

Regional plot of the FTO locus. Regional plot, generated by FUMA, for the *FTO* locus showing the top lead variant rs8047587 in purple in the age-adjusted meta-analysis (without 23andMe data, n=457,598 samples). A statistically significant association between the *FTO* variant and polycystic ovary syndrome (PCOS) was not observed after body mass index (BMI) adjustment (p=0.02). Each variant is color-coded based on the highest r^2 to one of the independent significant variants, if that is greater or equal to the user defined threshold ($r^2 \geq 0.6$). Other variants (i.e. below the user-defined r^2) are colored in grey. The top lead variants in genomic risk loci, lead variants and independent significant variants are circled in black and colored in dark-purple, purple and red, respectively.

Supplementary Figure 4

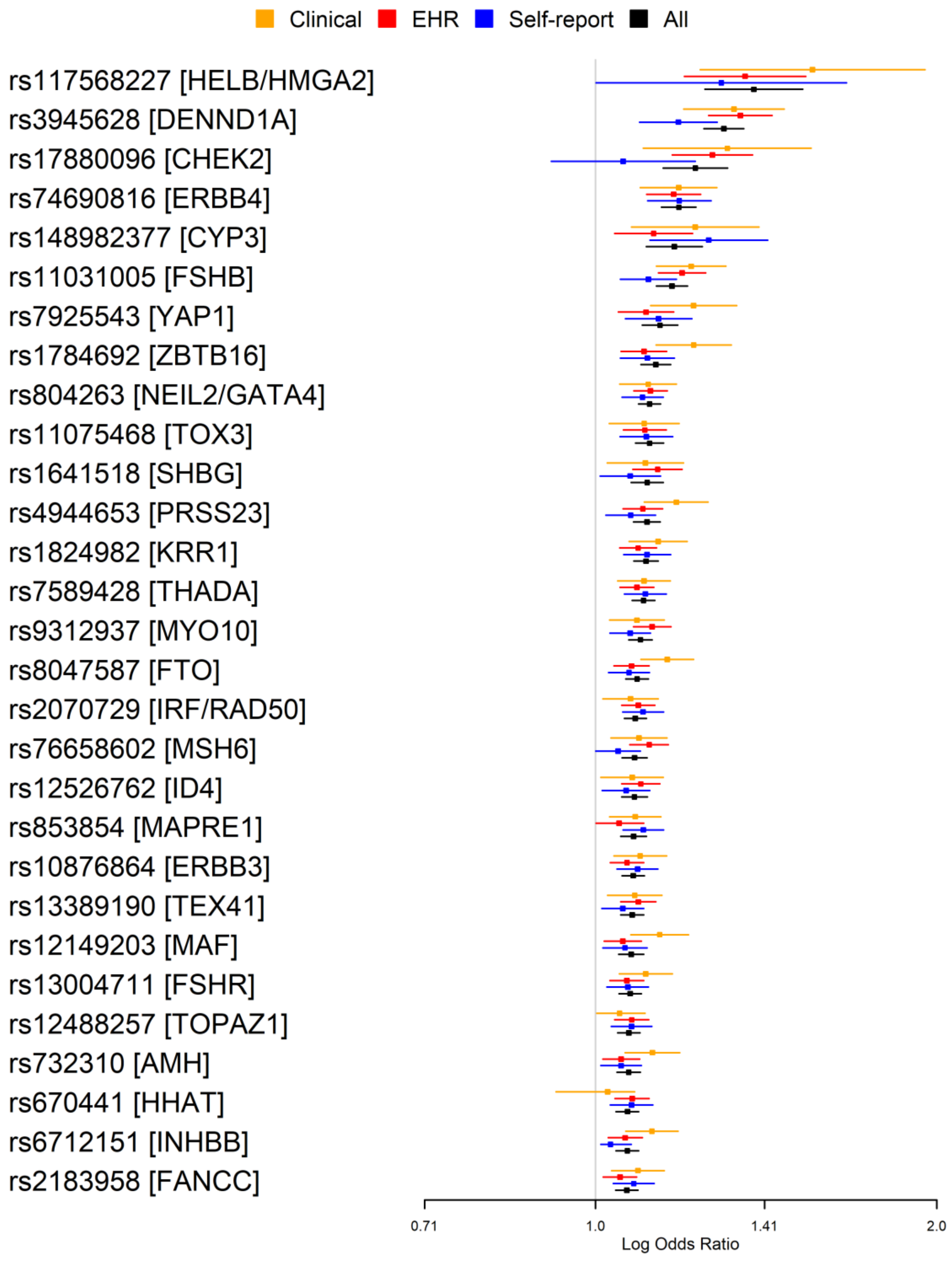

Forest plot of the 29 identified loci by source of polycystic ovary syndrome (PCOS) designation. Clinically defined (orange), electronic health record identified (EHR, red), self-report (blue) and all sources (black) are indicated. Error bars represent the 95% confidence intervals. Details on the sample size for each PCOS designation can be found in **Supplementary Table 1**.

Supplementary Figure 5

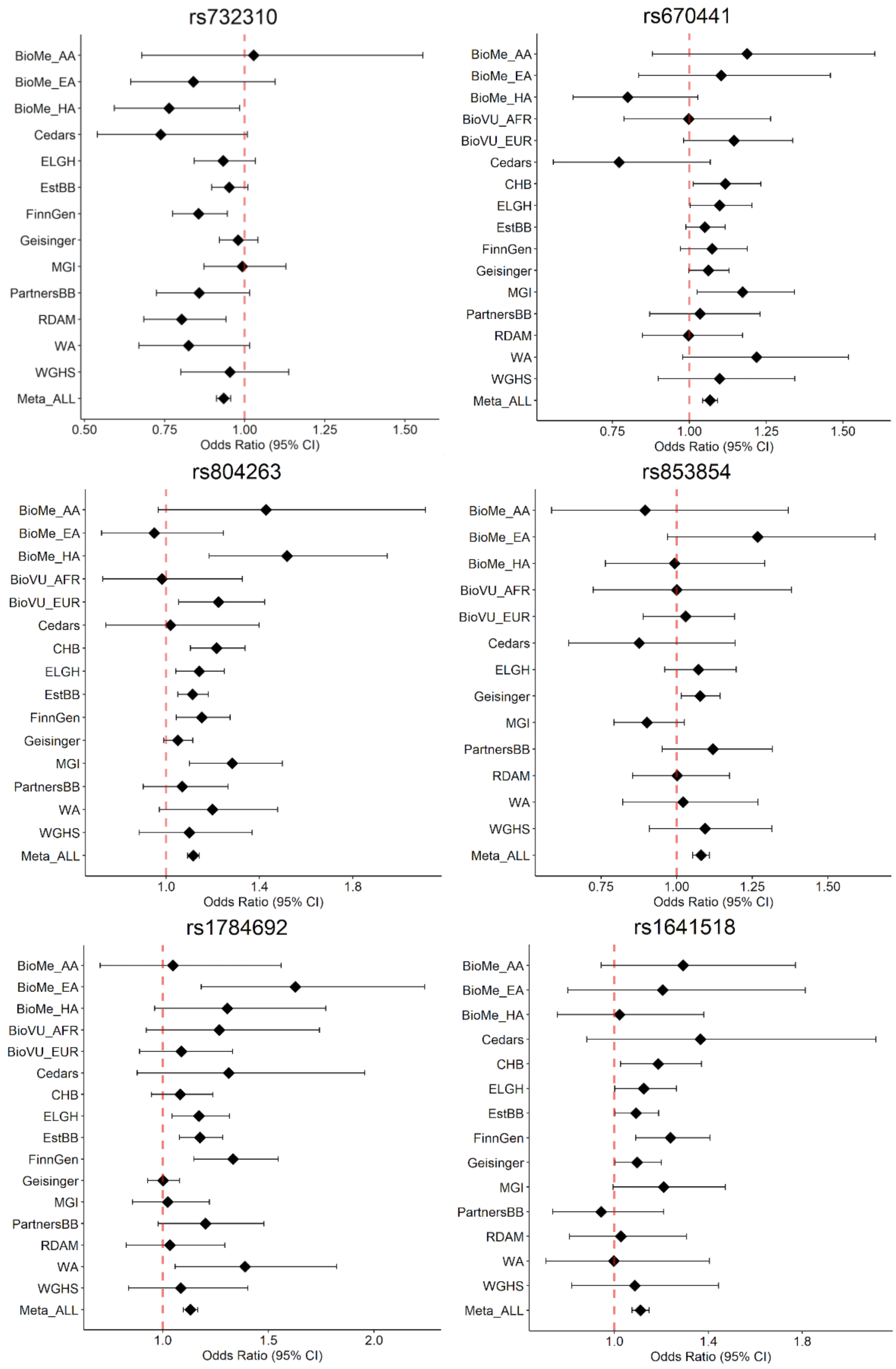

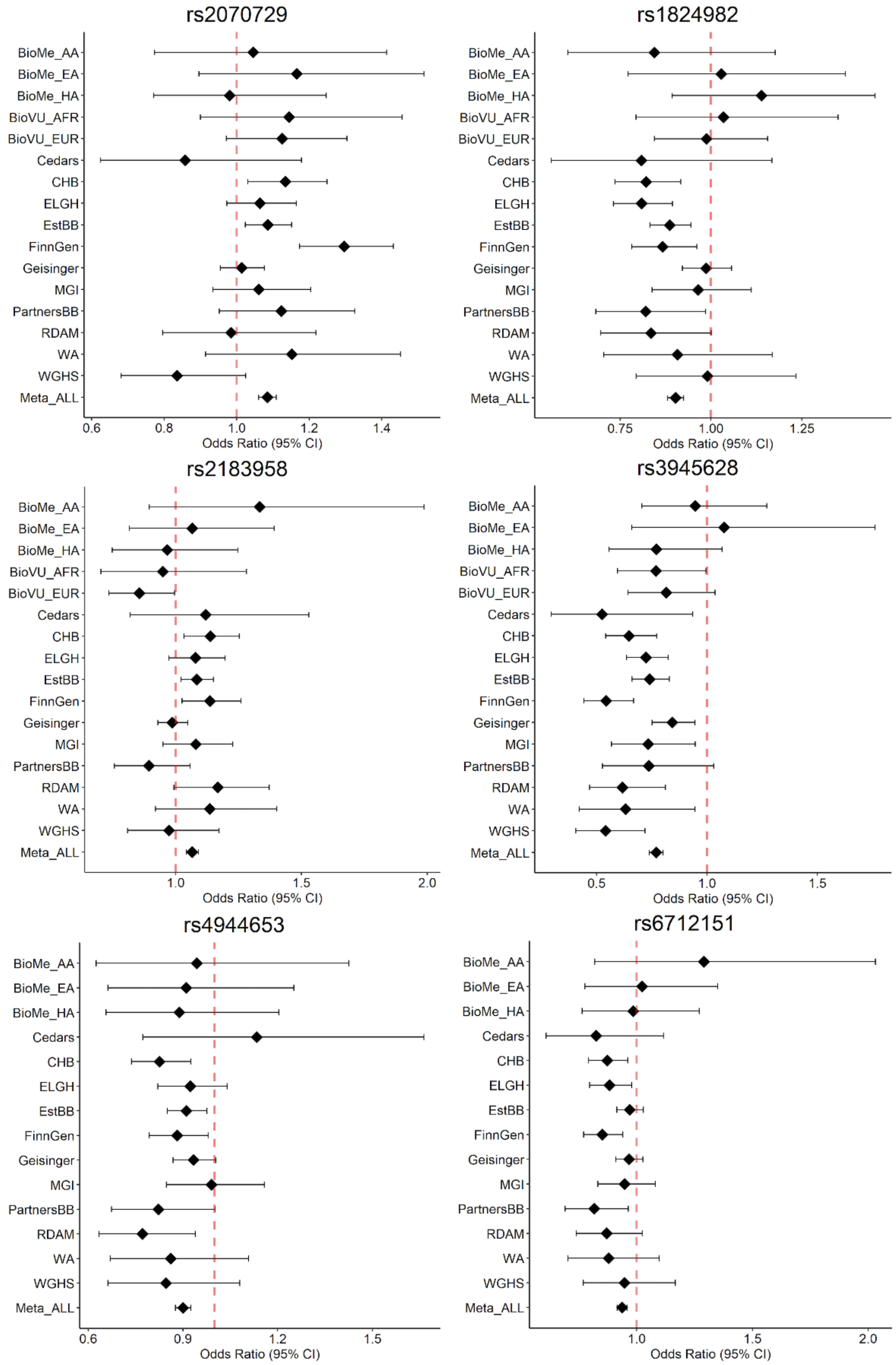
rs2070729
BioMe_AA
BioMe_EA
BioMe_HA
BioVU_AFR
BioVU_EUR
Cedars
CHB
ELGH
EstBB
FinnGen
Geisinger
MGI
PartnersBB
RDAM
WA
WGHS
Meta_ALL
0.6
0.8
1.0
1.2
1.4
Odds Ratio (95% CI)
rs1824982
BioMe_AA
BioMe_EA
BioMe_HA
BioVU_AFR
BioVU_EUR
Cedars
CHB
ELGH
EstBB
FinnGen
Geisinger
MGI
PartnersBB
RDAM
WA
WGHS
Meta_ALL
0.75
1.00
1.25
Odds Ratio (95% CI)
rs2183958
BioMe_AA
BioMe_EA
BioMe_HA
BioVU_AFR
BioVU_EUR
Cedars
CHB
ELGH
EstBB
FinnGen
Geisinger
MGI
PartnersBB
RDAM
WA
WGHS
Meta_ALL
1.0
1.5
2.0
Odds Ratio (95% CI)
rs3945628
BioMe_AA
BioMe_EA
BioMe_HA
BioVU_AFR
BioVU_EUR
Cedars
CHB
ELGH
EstBB
FinnGen
Geisinger
MGI
PartnersBB
RDAM
WA
WGHS
Meta_ALL
0.5
1.0
1.5
Odds Ratio (95% CI)
rs4944653
BioMe_AA
BioMe_EA
BioMe_HA
Cedars
CHB
ELGH
EstBB
FinnGen
Geisinger
MGI
PartnersBB
RDAM
WA
WGHS
Meta_ALL
0.6
0.9
1.2
1.5
Odds Ratio (95% CI)
rs6712151
BioMe_AA
BioMe_EA
BioMe_HA
Cedars
CHB
ELGH
EstBB
FinnGen
Geisinger
MGI
PartnersBB
RDAM
WA
WGHS
Meta_ALL
1.0
1.5
2.0
Odds Ratio (95% CI)

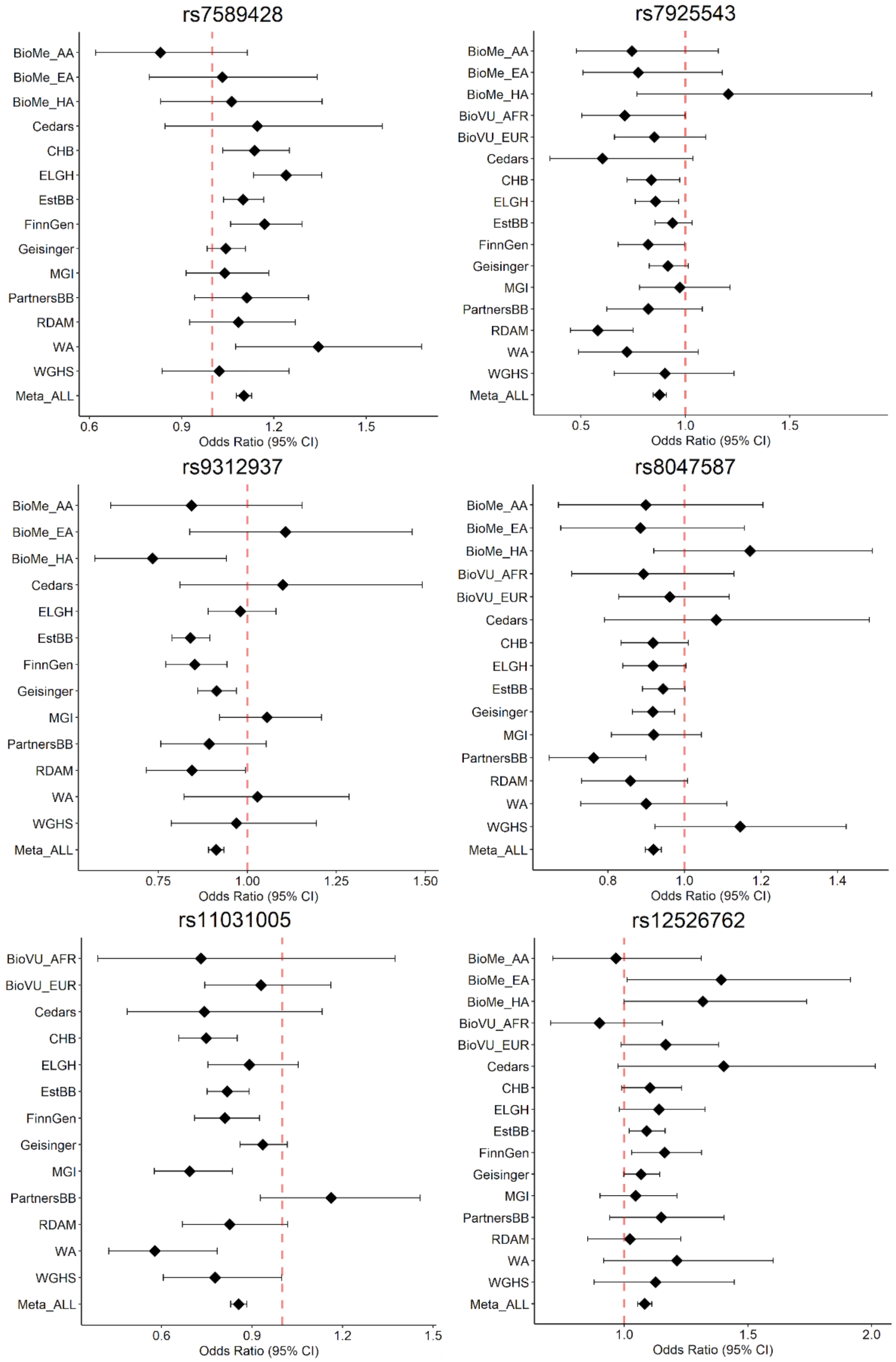
rs7589428
BioMe_AA
BioMe_EA
BioMe_HA
Cedars
CHB
ELGH
EstBB
FinnGen
Geisinger
MGI
PartnersBB
RDAM
WA
WGHS
Meta_ALL
0.6
0.9
1.2
1.5
Odds Ratio (95% CI)
rs7925543
BioMe_AA
BioMe_EA
BioMe_HA
BioVU_AFR
BioVU_EUR
Cedars
CHB
ELGH
EstBB
FinnGen
Geisinger
MGI
PartnersBB
RDAM
WA
WGHS
Meta_ALL
0.5
1.0
1.5
Odds Ratio (95% CI)
rs9312937
BioMe_AA
BioMe_EA
BioMe_HA
Cedars
ELGH
EstBB
FinnGen
Geisinger
MGI
PartnersBB
RDAM
WA
WGHS
Meta_ALL
0.75
1.00
1.25
1.50
Odds Ratio (95% CI)
rs8047587
BioMe_AA
BioMe_EA
BioMe_HA
BioVU_AFR
BioVU_EUR
Cedars
CHB
ELGH
EstBB
Geisinger
MGI
PartnersBB
RDAM
WA
WGHS
Meta_ALL
0.8
1.0
1.2
1.4
Odds Ratio (95% CI)
rs11031005
BioVU_AFR
BioVU_EUR
Cedars
CHB
ELGH
EstBB
FinnGen
Geisinger
MGI
PartnersBB
RDAM
WA
WGHS
Meta_ALL
0.6
0.9
1.2
1.5
Odds Ratio (95% CI)
rs12526762
BioMe_AA
BioMe_EA
BioMe_HA
BioVU_AFR
BioVU_EUR
Cedars
CHB
ELGH
EstBB
FinnGen
Geisinger
MGI
PartnersBB
RDAM
WA
WGHS
Meta_ALL
1.0
1.5
2.0
Odds Ratio (95% CI)

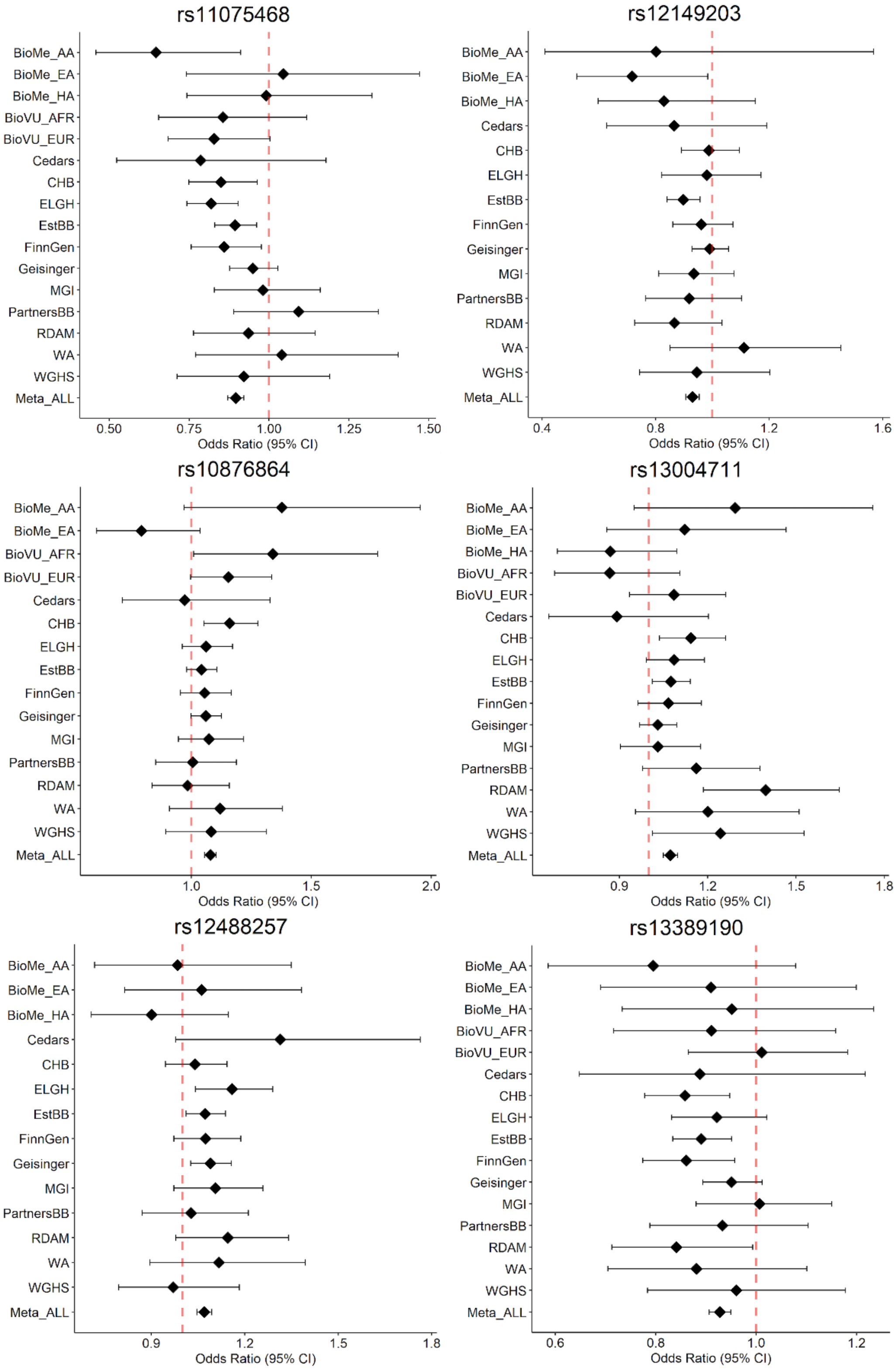
rs11075468
BioMe_AA
BioMe_EA
BioMe_HA
BioVU_AFR
BioVU_EUR
Cedars
CHB
ELGH
EstBB
FinnGen
Geisinger
MGI
PartnersBB
RDAM
WA
WGHS
Meta_ALL
0.50
0.75
1.00
1.25
1.50
Odds Ratio (95% CI)
rs12149203
BioMe_AA
BioMe_EA
BioMe_HA
Cedars
CHB
ELGH
EstBB
FinnGen
Geisinger
MGI
PartnersBB
RDAM
WA
WGHS
Meta_ALL
0.4
0.8
1.2
1.6
Odds Ratio (95% CI)
rs10876864
BioMe_AA
BioMe_EA
BioVU_AFR
BioVU_EUR
Cedars
CHB
ELGH
EstBB
FinnGen
Geisinger
MGI
PartnersBB
RDAM
WA
WGHS
Meta_ALL
1.0
1.5
2.0
Odds Ratio (95% CI)
rs13004711
BioMe_AA
BioMe_EA
BioMe_HA
BioVU_AFR
BioVU_EUR
Cedars
CHB
ELGH
EstBB
FinnGen
Geisinger
MGI
PartnersBB
RDAM
WA
WGHS
Meta_ALL
0.9
1.2
1.5
1.8
Odds Ratio (95% CI)
rs12488257
BioMe_AA
BioMe_EA
BioMe_HA
Cedars
CHB
ELGH
EstBB
FinnGen
Geisinger
MGI
PartnersBB
RDAM
WA
WGHS
Meta_ALL
0.9
1.2
1.5
1.8
Odds Ratio (95% CI)
rs13389190
BioMe_AA
BioMe_EA
BioMe_HA
BioVU_AFR
BioVU_EUR
Cedars
CHB
ELGH
EstBB
FinnGen
Geisinger
MGI
PartnersBB
RDAM
WA
WGHS
Meta_ALL
0.6
0.8
1.0
1.2
Odds Ratio (95% CI)

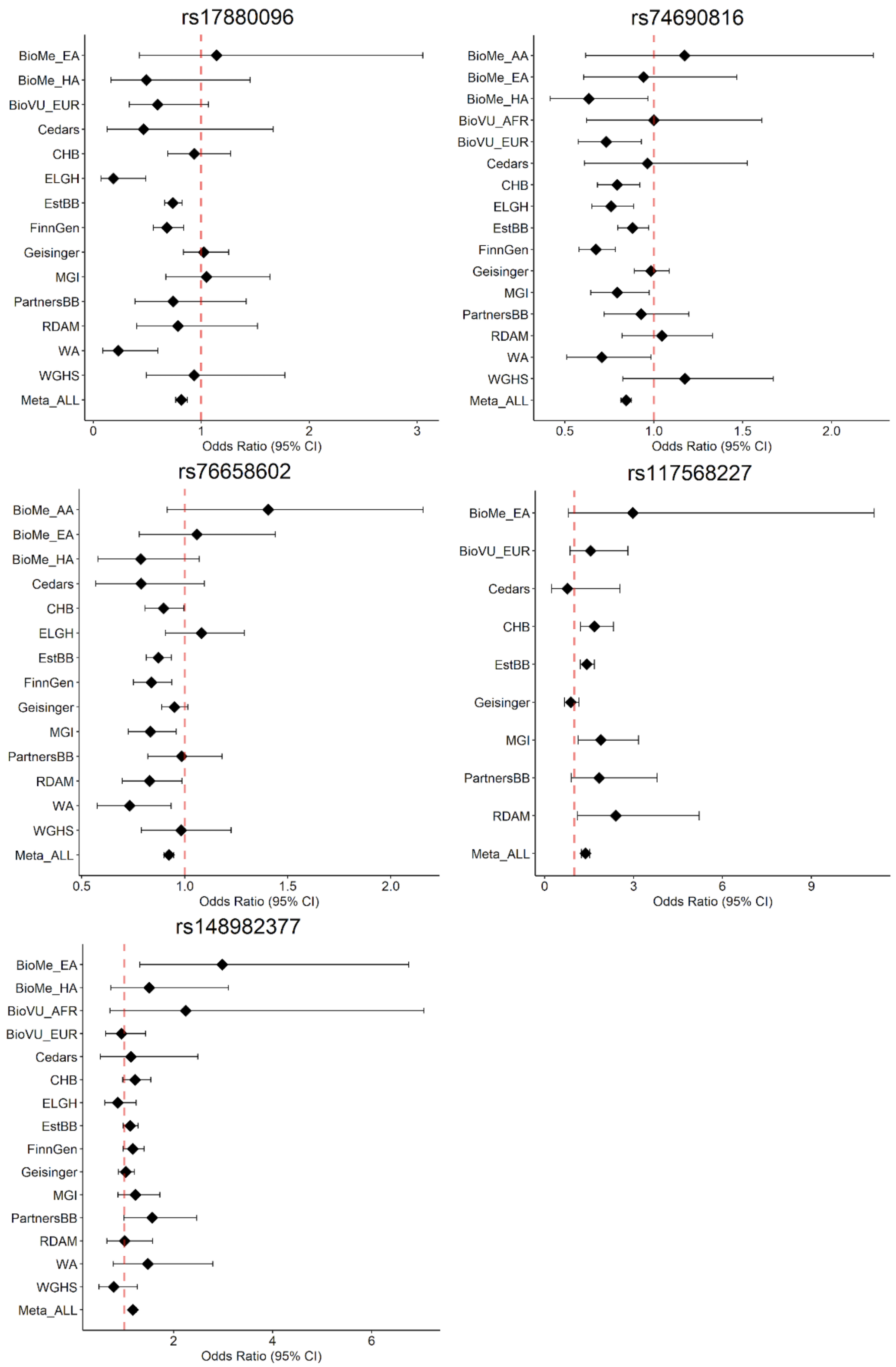

Forest plots for the top 29 polycystic ovary syndrome (PCOS) associations for each study cohort. The effect sizes with the 95% confidence intervals shown as error bars are indicated for each of the 29 variants (individual panels) in the 13 studies stratified by ancestry in the age-adjusted meta-analysis. Details on the sample size for each individual cohort can be found in **Supplementary Table 1.**

Supplementary Figure 6

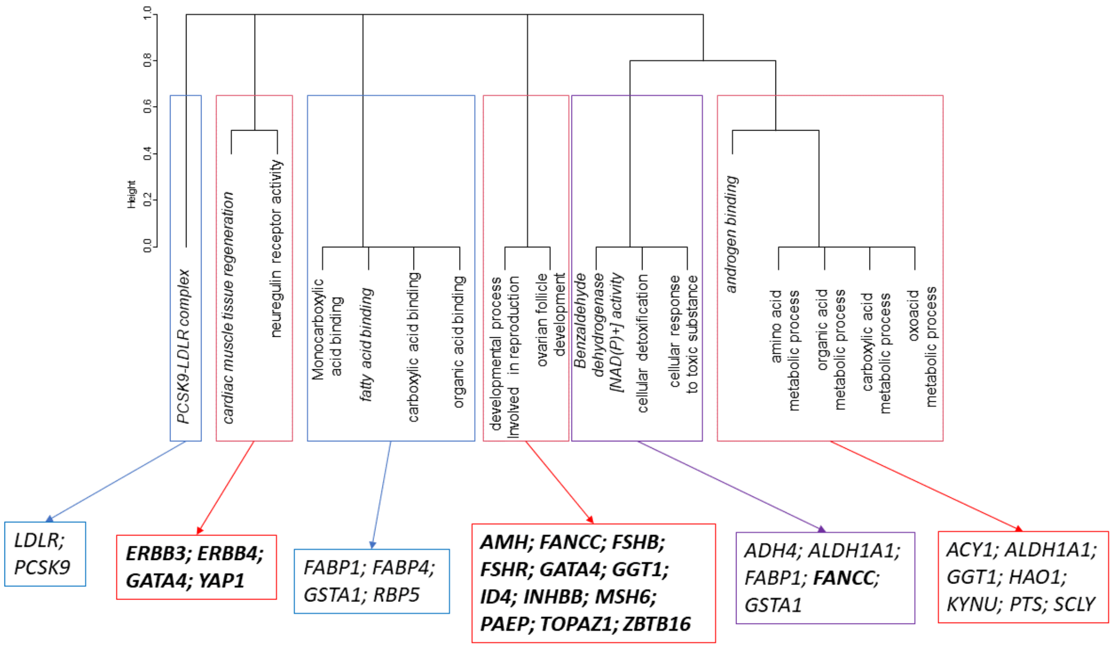

Clustered Pathways based on a combined list of associated genetic loci and proteins associated with a diagnosis coded as E28. Clusters were generated based on similarity of the intersection between pathways and polycystic ovary syndrome (PCOS) genes. Clusters driven by genetic associations are shown in red, with genes in bold. Those driven by proteins are shown in blue, and the one cluster with interactions covering both discovery sets is shown in purple. In each case the strongest associated pathway in each cluster is in italic.

Supplementary Figure 7

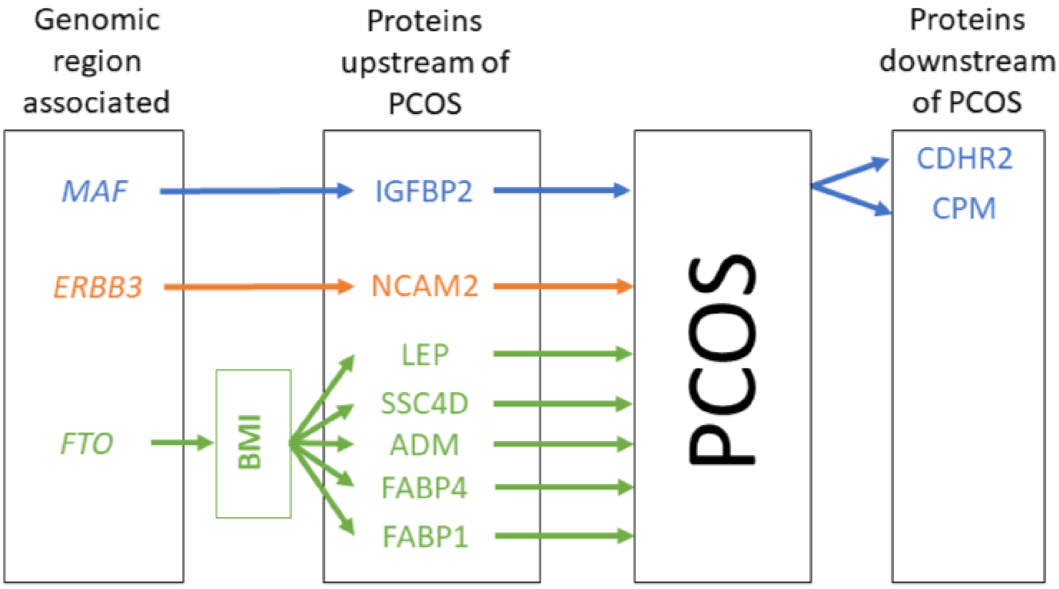

Map of the associations between polycystic ovary syndrome (PCOS) and E28 (ovarian dysfunction) associated proteins. Using PCOS as the base phenotype, proteins immediately upstream and downstream of PCOS are indicated. If the variant was associated with greater explained variance in protein levels compared to PCOS, it was likely that protein played a role in PCOS and was therefore situated upstream. Conversely, if the greater explained variance came from the PCOS designation, it was likely affected by PCOS, itself. The genomic regions affecting protein levels were direct, except in the case of FTO, in which protein associations were mediated by BMI.

Supplementary Figure 8

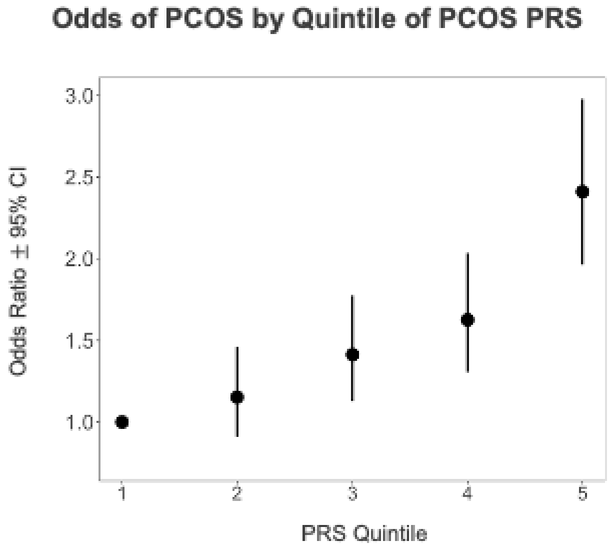

Association of the polycystic ovary syndrome-polygenic risk score (PCOS-PRS) with PCOS in the UK Biobank. The sample includes 1,003 cases and 205,849 controls. ($P=9\times10^{-27}$, based on 1,003 cases and 205,849 controls). Each increasing PCOS-PRS quintile was associated with a greater odds of PCOS. Error bars represent the 95% confidence intervals.

Supplementary Figure 9

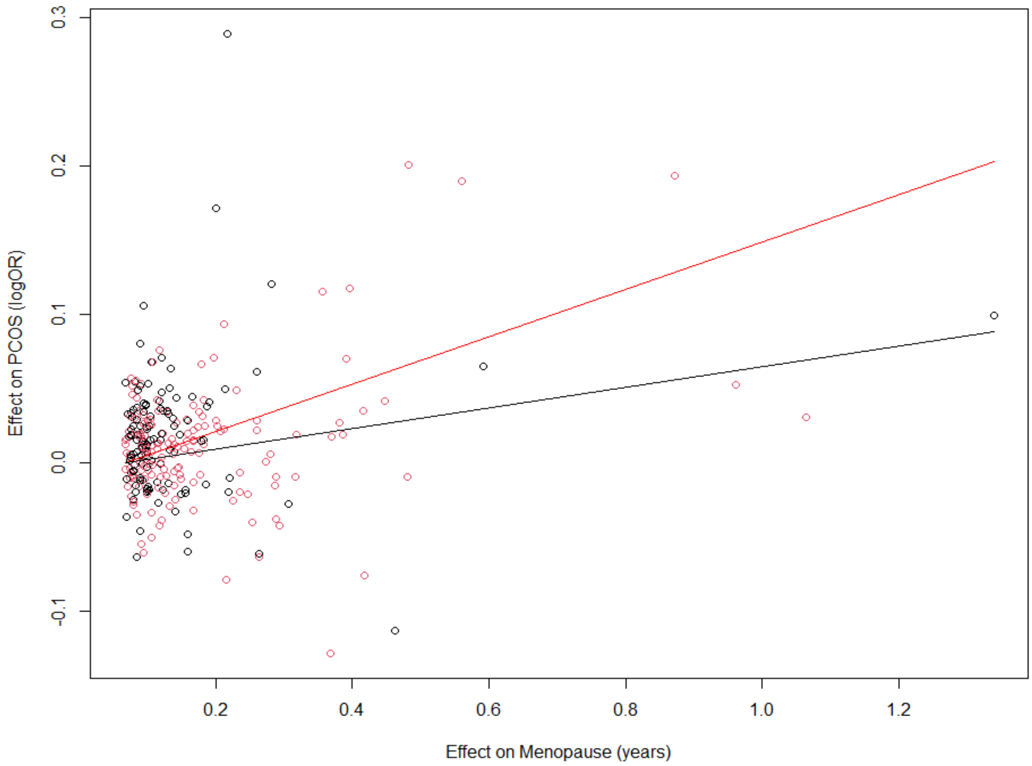

Relationship between DNA damage repair (DDR) genes and non-DDR genes in relation to effect on polycystic ovary syndrome and menopause. Comparison of the inverse weighted variance - Mendelian randomisation (IVW-MR) split by DDR related menopause genes and (black) non-DDR related menopause variants (red). P-value for the difference between estimates = 1.5×10^{-6}.

Supplementary Table

Supplementary Table 25. International Classification of Disease (ICD) codes used to identify women with PCOS in electronic medical records.

Diagnosis	ICD8	ICD9	ICD10
**INCLUSION DIAGNOSES**			
PCOS	256.9	256.4	E28.2
Irregular Menses	626	626.X	N92
Hirsutism	704	704.1	L68.0
**EXCLUSION DIAGNOSES**			
Premature Ovarian Failure	256.1, 256.3	256.3, 256.31	E28.39
Cushing Syndrome	255, 258	255.0, 258.0	E24
Hypothalamic amenorrhea	256.1	256.8	E23.3
Congenital Adrenal Hyperplasia	255	255.2	E25
Eating disorder	306.5	307.1x, 307.5x	F50.9, F50.2, F50.01
Chronic opioid use	304	304.X	F11
Fibroids	218	654.1X, 218.9	O34.1, D25.9
Pituitary adenoma	226.2	253.X	D352
Pituitary hypersecretion	253	253.X	E22
Hyperprolactinemia	253	253.1	E22
Ovarian tumor	183	239.5	C56
Benign neoplasm of the ovary (Leydig cell tumor, hilus cell tumor)	220	220	D27.9
Turner syndrome	759.5	758.6	Q96
Galactorrhea	253	253.1	N64.3
Suprarenal tumor	226.0	227.0, 194.0	C74

Supplementary Note

Supplementary Methods

Subjects

The first stage of the meta-analysis included 11,653 cases and 423,614 controls from 16 data sets from studies of European, African American, Hispanic American and South Asian descent. Cases were diagnosed with PCOS based on NIH or Rotterdam Criteria, by self-report or using International Classification of Disease codes for PCOS or irregular menses and hirsutism (**Supplementary Table 1**). The NIH criteria require the presence of both OD and clinical and/or biochemical HA for a diagnosis of PCOS[1]. The Rotterdam criteria require two out of three features 1) OD defined by oligo- or amenorrhea (chronic menstrual cycle interval >35 days in all cohorts), 2) clinical and/or biochemical hyperandrogenism (HA) and/or 3) PCOM for a diagnosis of PCOS[1]. Non-NIH Rotterdam was defined by OD and PCOM or clinical and/or biochemical hyperandrogenism (HA) and PCOM. This was combined with previous published data. Self-reported female cases from research participants in the 23andMe, Inc. (San Francisco, CA, USA) cohort either responded “yes” to the question “Have you ever been diagnosed with polycystic ovary syndrome?” or indicated a diagnosis of PCOS when asked about fertility (“Have you ever been diagnosed with PCOS?” or “What was your diagnosis? Please check all that apply.” Answer=PCOS), hair loss in men or women (“Have you been diagnosed with any of the following? Please check all that apply.” Answer=PCOS) or research question (“Have you ever been diagnosed with PCOS?”).[2] 23andMe controls were female, only.

HA was defined as hirsutism and quantified by the Ferriman-Gallwey (FG) score. The FG score assesses terminal hair growth in a male pattern in females, and a score above the upper limit of normal controls (>8) is considered hirsutism.[3] Hyperandrogenemia was defined as testosterone, androstenedione or DHEAS greater than the 95% confidence limits in control subjects in the individual population. OD was defined as cycle interval <21 or >35 days. PCOM was defined as 12 or more follicles of 2-9 mm in at least one ovary or an ovarian volume >10 mL.[4] The quantitative PCOS traits included levels of total testosterone (T), follicle-stimulating hormone (FSH), and luteinizing hormone (LH) and ovarian volume. An overview of the diagnostic criteria is provided in **Supplementary Table 25**.

Summary-data-based Mendelian Randomisation (SMR) Analysis

In addition to the SMR analyses performed as part of the GWAS-to-Genes pipeline, we performed an integrative analysis on the PCOS GWAS meta-analysis results and expression quantitative trait locus (eQTL) association results for PCOS-relevant GTEx tissues and plasma protein QTL (pQTL) data using the SMR software[5,6]. This package utilises Mendelian randomisation principles to assess for association between gene/protein expression and a trait due to a shared genetic variant (pleiotropy). Dual association signals in the GWAS and eQTL/pQTL datasets are identified by testing for association between gene expression and the trait of interest at the top eQTL/pQTL variant for each gene. The software also performs a heterogeneity in dependent instruments (HEIDI) test, which compares the association signals for nearby co-inherited markers in the GWAS and eQTL/pQTL datasets. If heterogeneity exists

in the association profiles of the two datasets, as indicated by a significant HEIDI test result, the association signals present in each dataset are considered less likely to be driven by the same causal variant. Genes with at least 1 *cis*-eQTL/pQTL association significant at $P<5\times10^{-8}$ were included in the SMR analysis, with linkage disequilibrium data from the 1000 Genomes Project phase 3 dataset used for the HEIDI test. The SMR analysis was performed using the all-ancestries age-adjusted (not including 23andMe) PCOS GWAS meta-analysis dataset, plasma pQTL results from the Atherosclerosis Risk in Communities (ARIC) study[7] and the GTEx V7 eQTL datasets[8] for the following PCOS-relevant tissues: adipose subcutaneous, adipose visceral omentum, adrenal gland, ovary, pancreas, pituitary and testis. Correction for multiple testing was performed for each tissue analysed using the Benjamini-Hochberg procedure, with a conservative significance threshold of P<0.05 used for the HEIDI test (P_{HEIDI}) as an indicator of heterogeneity.

Polygenic Risk Score (PRS) Phenotype Definitions in the UK Biobank

1) Coronary artery disease (CAD)[9]
Self-report: Age heart attack diagnosed

Ischemic Heart diseases
ICD-10 I21-I25.X
ICD-9 410-412.X

Coronary Revascularization:
OPCS4 K40,41,45,49,50,75

Date of MI: Combination of 5 outcome data fields

2) Type 2 diabetes (T2D)[10,11]
Baseline Visit Algorithm:
Diabetes diagnosis
Medication use
Age at diagnosis

Self-reported diagnosis

Repeat Visits
Diabetes diagnosis
Medication information

Exclusions
Age of diabetes <40 years
Controls >=55 years

3) Breast Cancer
Type of cancer:
ICD-9: 174X

ICD-10: C50.X
Cancer code, self-reported
1002

Hormonal cancer phenotypes: Breast cancer was based on self-report and/or ICD-9 codes 174.0-174.9 and ICD-10 codes C50.0-C50.9 in hospitalisation records.

4) Childlessness
Cases

Women: Field code 2734 (number of live births) = 0
Men: Field code 2405 (number of children fathered) = 0

Controls:
Women: Field code 2734 (number of live births) neither 0 or NA
Men: Field code 2405 (number of children fathered) neither 0 or NA

5) Bipolar/depression status[12]
Derived field 20126

Mental health phenotypes: Depression was defined by self-report of current and previous depressive symptoms and items from a Patient Health Questionnaire and on seeking evaluation for mental health, and controls were defined as individuals with no history of bipolar disorder or depression.

6) Asthma
Cases
1. Field code 6152_8 (doctor diagnosed asthma)
2. ICD-10: J45 (asthma)/J46 (severe asthma)
3. Self-reported asthma

Controls:
1. Free from field code 6152_8 (doctor diagnosed asthma)
2. Free from field code 6152_9 (doctor diagnosed allergic diseases)
3. Free from ICD10 J45/J46/J30 (hay fever)/L20 (dermatitis and eczema)
4. Free from self-reported asthma/hay fever/eczema/allergy/ allergy to house dust mite (HDM)

Asthma was defined by a composite of self-reported diagnosis of asthma, self-reported diagnosis of asthma by a doctor, and/or ICD-10 codes J45.0, J45.1, J45.8, J45.9, J46 from hospitalisation records. Controls were defined by individuals free from a diagnosis of hay fever (ICD-10 J30.0-J30.4), dermatitis and eczema (ICD-10 L20.8, L20.9), or self-reported diagnosis of asthma or other allergic disorders (hay fever, eczema, allergy, and allergy to house dust mite).

Supplementary Results

SMR analysis in PCOS-relevant tissues

After correction for multiple testing, 26 significant associations were identified between the PCOS GWAS meta-analysis results and the eQTL datasets, including five for adipose subcutaneous, six for adipose visceral omentum, one for adrenal gland, two for ovary, three for pancreas, three for pituitary and six for testis. Of these, 18 demonstrated evidence of pleiotropic effects on gene expression and PCOS using the HEIDI test, with 12 located in genome-wide significant loci. These tissues represent a range of important organs in the aetiology of PCOS. Some genes showed particularly strong evidence for an association with PCOS. The *RPS26* gene, which encodes a ribosomal protein, demonstrated significant pleiotropic associations in all tissues analysed except for the adrenal gland. In each instance, expression of RPS26 was found to be positively associated with PCOS risk (β_{SMR}=0.06). In some cases, the SMR analyses revealed more complex biological processes, with gene *ARL14EP* prioritised on chromosome 11, despite strong *a-priori* evidence that this variant is acting through the nearby *FSHB*. Sometimes it provided better resolution of established signals, with the variant previously tagged as *GATA2* showing strong evidence of an effect via *NEIL2*. The *NEIL2* gene, encoding a DNA glycosylase enzyme, demonstrated significant pleiotropic associations in the adipose visceral omentum, ovary and pituitary tissues. In each of these tissues, expression of *NEIL2* was found to be negatively associated with PCOS risk (β_{SMR}=-0.2 to -0.4). Finally, we were also able to show that increased expression of the established age-at-menopause gene *MSH6*[6] in the testis is positively associated with PCOS risk (β_{SMR}=0.3). No significant associations were identified between the PCOS GWAS meta-analysis results and the pQTL data after correction for multiple testing.

Analysis of previously reported loci

We assessed 10 PCOS signals previously reported in East Asian women[13] (**Supplementary Table 5**). Of these, all but three (variants near *C9orf3*, *INSR* and *SUMO1P1*) had statistically significant associations in our study (*P*<0.005). The *SUMO1P1* minor allele was more common and had a relatively smaller effect size (OR=1.03) with borderline evidence for association (*P*=0.0058) in our data. The variant at *C9orf3* had a frequency in our data of approximately half that in the Chinese data, whereas the variant in *INSR,* coding for the insulin receptor, was more common in our data. Although rare deleterious variants in *INSR* cause a severe PCOS phenocopy[14], previous studies have not demonstrated an association at the *INSR* locus in women of European ancestry[15]. The relative frequency of the *INSR* variant suggests that there might be a gene-environment interaction that explains the different findings between these two studies or that the Han Chinese variant tags a haplotype that is not present in Europeans.

Fine-mapping of PCOS signals

Fine-mapping of the identified PCOS loci was performed using a linkage disequilibrium (LD) reference based on European ancestry women in the MyCode/DiscovEHR and, separately, using the BioVU study (**Supplementary Tables 6 and 7**). At seven of the loci, both analyses resolved to the same sentinel variant. At *LLPH*, rs117568227 was the only variant in the 95% credible set [Posterior Probability (PP)>0.99] in both studies, and at *YAP1,* rs7925543 was the

only variant (PP=0.64 in MyCode/DiscovEHR and 0.79 in BioVU). Another 10 PCOS loci resolved to 20 or fewer variants across the two analyses.

Protein analysis

Having identified these 299 proteins, we then assessed if any showed evidence of association with a diagnosis of ICD E28 (ovarian dysfunction), based on a threshold of $P<1.7\times10^{-4}$ (0.05/299 proteins analysed). This two-stage approach resulted in nine variant-protein pairs. Three proteins were associated with the PCOS signal at *MAF* (CDHR2, IGFBP2, CPM), one with *ERBB3* (NCAM2) and five with *FTO* (ADM, FABP1, FABP4, LEP and SSC4D) (**Supplementary Table 20**). The protein associations seen at the *FTO* locus are likely a result of the adiposity effect of this locus, either through effects on BMI or shared biological pathways. This is further highlighted by the fact that, of these proteins, FABP4 and LEP have recently been shown to be linked to aging specifically in adipose tissue[16], which would suggest that changes in the levels are due to a primarily BMI-related effect. To understand the direction between individual plasma proteins and PCOS, we compared the variance in each trait explained by its respective PCOS signal (variance in BMI was also compared for those proteins associated with *FTO*) (**Supplementary Figure 7**). The approach is based on the assumption that if a variant was associated with greater explained variance in protein levels compared to PCOS, it was unlikely that protein was affected as a result of PCOS. This logic is similar to that used as the basis of Steiger filtering[17] in Mendelian Randomisation. While that approach is useful for understanding the relationship between two complex phenotypes with many SNPs, here our pQTLs include one variant only rather than assessing two complex phenotypes with many SNPs. All five *FTO* associated proteins, NCAM2 associated with *ERBB3*, and IGFBP2 associated with *MAF*, appeared to be upstream (i.e. more likely to be determinants, or common soil) of PCOS. The two other proteins associated with *MAF,* CDHR2 and CPM, may have their levels altered as a consequence of PCOS.

Additional Cohort-specific Details

FinnGen

We want to acknowledge the participants and investigators of the FinnGen study. The FinnGen project is funded by two grants from Business Finland (HUS 4685/31/2016 and UH 4386/31/2016) and the following industry partners: AbbVie Inc., AstraZeneca UK Ltd, Biogen MA Inc., Bristol Myers Squibb Inc. (and Celgene Corporation & Celgene International II Sàrl), Genentech Inc., Merck Sharp & Dohme LCC, Pfizer Inc., GlaxoSmithKline Intellectual Property Development Ltd., Sanofi US Services Inc., Maze Therapeutics Inc., Johnson&Johnson Innovative Medicine Inc., Novartis AG, Boehringer Ingelheim International GmbH and Bayer AG. Following biobanks are acknowledged for delivering biobank samples to FinnGen: Auria Biobank (www.auria.fi/biopankki), THL Biobank (www.thl.fi/biobank), Helsinki Biobank (www.helsinginbiopankki.fi), Biobank Borealis of Northern Finland (https://www.ppshp.fi/Tutkimus-ja-opetus/Biopankki/Pages/Biobank-Borealis-briefly-in-English.aspx), Finnish Clinical Biobank Tampere (www.tays.fi/en-US/Research_and_development/Finnish_Clinical_Biobank_Tampere), Biobank of Eastern Finland (www.ita-suomenbiopankki.fi/en), Central Finland Biobank (www.ksshp.fi/fi-FI/Potilaalle/Biopankki), Finnish Red Cross Blood Service Biobank (www.veripalvelu.fi/verenluovutus/biopankkitoiminta), Terveystalo Biobank (www.terveystalo.com/fi/Yritystietoa/Terveystalo-Biopankki/Biopankki/) and Arctic Biobank (https://www.oulu.fi/en/university/faculties-and-units/faculty-medicine/northern-finland-birth-cohorts-and-arctic-biobank). All Finnish Biobanks are members of BBMRI.fi infrastructure (https://www.bbmri-eric.eu/national-nodes/finland/). Finnish Biobank Cooperative -FINBB (https://finbb.fi/) is the coordinator of BBMRI-ERIC operations in Finland. The Finnish biobank data can be accessed through the Fingenious® services (https://site.fingenious.fi/en/) managed by FINBB.

Estonian BioBank

The Estonian Biobank (EstBB) is a population-based biobank with over 200,000 participants, currently including approximately 135,000 women (20% of the female Estonian population). The 150K data freeze was used for the analyses described in this paper. All biobank participants have signed a broad informed consent form. Individuals with PCOS were identified using the ICD-10 code E28.2, and all female biobank participants who did not have this diagnosis served as controls. This included a total of 2,812 cases and 89,230 controls. Information on the ICD codes was obtained via regular linking with the national Health Insurance Fund and other relevant databases.

Estonian Biobank research team (Affiliation: Estonian Genome Centre, Institute of Genomics, University of Tartu / e-mail: EstBBresearch@ut.ee): Andres Metspalu, Lili Milani, Tõnu Esko, Reedik Mägi, Mari Nelis and Georgi Hudjashov.

Leitsalu L, Haller T, Esko T, Tammesoo ML, Alavere H, Snieder H, et al. Cohort Profile: Estonian Biobank of the Estonian Genome Center, University of Tartu. Int J Epidemiol 2015 Aug;44(4):1137- 1147.

Dutch Cohort

Data from the Dutch Cohort consists of the Rotterdam PCOS Cohort, with PCOS cases diagnosed in Erasmus Medical Centre, Rotterdam by thorough standardized screening[18]. Controls are provided by the Lifelines Cohort study[19]. The lifelines initiative has been made possible by subsidy from the Dutch Ministry of Health, Welfare and Sport, the Dutch Ministry of Economic Affairs, the University Medical Center Groningen (UMCG), Groningen University and the Provinces in the North of the Netherlands (Drenthe, Friesland, Groningen).Lifelines is a multi-disciplinary prospective population-based cohort study examining in a unique three-generation design the health and health-related behaviours of 167,729 persons living in the North of the Netherlands. It employs a broad range of investigative procedures in assessing the biomedical, sociodemographic, behavioural, physical and physiological factors which contribute to the health and disease of the general population, with a special focus on multi-morbidity and complex genetics.

Genes and Health

Genes & Health is/has recently been core-funded by Wellcome (WT102627, WT210561), the Medical Research Council (UK) (M009017, MR/X009777/1), Higher Education Funding Council for England Catalyst, Barts Charity (845/1796), Health Data Research UK (for London substantive site), and research delivery support from the NHS National Institute for Health Research Clinical Research Network (North Thames). Genes & Health is/has recently been funded by Alnylam Pharmaceuticals, Genomics PLC; and a Life Sciences Industry Consortium of Astra Zeneca PLC, Bristol-Myers Squibb Company, GlaxoSmithKline Research and Development Limited, Maze Therapeutics Inc, Merck Sharp & Dohme LLC, Novo Nordisk A/S, Pfizer Inc, Takeda Development Centre Americas Inc.

We thank Social Action for Health, Centre of The Cell, members of our Community Advisory Group, and staff who have recruited and collected data from volunteers. We thank the NIHR National Biosample Centre (UK Biocentre), the Social Genetic & Developmental Psychiatry Centre (King's College London), Wellcome Sanger Institute, and Broad Institute for sample processing, genotyping, sequencing and variant annotation. We thank: Barts Health NHS Trust, NHS Clinical Commissioning Groups (City and Hackney, Waltham Forest, Tower Hamlets, Newham, Redbridge, Havering, Barking and Dagenham), East London NHS Foundation Trust, Bradford Teaching Hospitals NHS Foundation Trust, Public Health England (especially David Wyllie), Discovery Data Service/Endeavour Health Charitable Trust (especially David Stables), NHS Digital - for GDPR-compliant data sharing backed by individual written informed consent. Most of all we thank all of the volunteers participating in Genes & Health.

Danish Blood Donors Study (DBDS) Genomic Consortium

Karina Banasik[1], Jakob Bay[2], Jens Kjaergaard Boldsen[5], Thosten Brodersen[2], Soren Brunak [1], Kristoffer Burgdorf [1], Mona Ameri Chalmer[3], Maria Didriksen[7], Khoa Manh Dinh [5], Joseph Dowsett[7], Bjarke Feenstra [7,8], Frank Geller [7,8], Daniel Gudbjartsson [4], Thomas Folkmann Hansen[3], Lotte Hindhede [5], Henrik Hjalgrim[14,15], Rikke Louise Jacobsen [7], Gregor Jemec[10], Bertram Dalskov Kjerulff[5], Lisette Kogelman[3], Margit Anita Horup Larsen [7], Ioannis

Louloudis[1], Agnete Lundgaard [1], Susan Mikkelsen[5], Ioanna Nissen [7], Alexander Pil Henriksen[1], Palle Duun Rohde[11], Klaus Rostgaard[14,15], Michael Schwinn [7], Kari Stefansson[4], Hreinn Stefansson [4], Erik Sorensen [7], Unnur Thorsteinsdottir [4], Lise Wegner Thorner[7], Thomas Werge[12,13], Mette Nyegaard[11], Sisse R. Ostrowski [7], Ole B.V. Pedersen[2], Christina Mikkelsen [7], Christian Erikstrup [5,6], Katrine Kaspersen[5], Mie T. Bruun[9], Bitten Aagaard[5], Henrik Ullum[16], David Westergaard[1]

1 Novo Nordisk Foundation Center for Protein Research, Faculty of Health and Medical Sciences, University of Copenhagen, Copenhagen, Denmark
2 Department of Clinical Immunology, Zealand University Hospital, Køge, Denmark
3 Danish Headache Center, Department of Neurology, Copenhagen University Hospital, Rigshospitalet-Glostrup, Copenhagen, Denmark
4 deCODE Genetics, Reykjavik, Iceland
5 Department of Clinical Immunology, Aalborg University Hospital, Aalborg, Denmark
6 Department of Clinical Medicine, Health, Aarhus University, Aarhus, Denmark
7 Department of Clinical Immunology, Copenhagen University Hospital, Rigshospitalet, Copenhagen, Denmark
8 Department of Epidemiology Research, Statens Serum Institut, Copenhagen, Denmark
9 Department of Clinical Immunology, Odense University Hospital, Odense, Denmark
10 Department of Dermatology, Zealand University hospital, Roskilde, Denmark
11 Department of Health Science and Technology, Faculty of Medicine, Aalborg University, Aalborg, Denmark
12 Institute of Biological Psychiatry, Mental Health Centre, Sct. Hans, Copenhagen University Hospital, Roskilde, Denmark
13 Department of Clinical Medicine, Faculty of Health and Medical Sciences, University of Copenhagen, Copenhagen, Denmark
14 Danish Cancer Society Research Center, Copenhagen, Denmark
15 Department of Epidemiology Research, Statens Serum Institut, Copenhagen, Denmark
16 Statens Serum Institut, Copenhagen, Denmark

Other Funding and Acknowledgements

Steering Committee: We thank the steering committee of the International PCOS Consortium. Members included Unnur Styrkarsdottir, John R. B. Perry and Felix Day, Andrea Dunaif, Joop Laven, Steve Franks, Cecilia M. Lindgren and Corrine K. Welt.

Funding: The WGHS is supported by the NHLBI (HL043851 and HL080467) and the NCI (CA047988 and UM1CA182913). This work has been supported by F32 HD103317, K08 HD110723 from the National Institute of Child Health and Human Development, 23CDA1054471 from the American Heart Association, Pediatric Endocrine Society Clinical Scholar Award and Boston Children's Hospital Office of Faculty Development Career Development Fellowship (JZ), MATER Marie Sklodowska-Curie which received funding from the European Union's Horizon 2020 research and innovation program under grant agreement No. 813707 (NPG), MRC grant MC_U106179472 YZ, KAK, FRD, KKO, JRBP), Samuel Oschin Comprehensive Cancer Institute Developmental Funds, Center for Bioinformatics and Functional Genomics and Department of Biomedical Sciences Developmental Funds (MRJ), Novo Nordisk Foundation (grants NNF17OC0027594 and NNF14CC0001 (KB and SB), A.P.

Moller Foundation (DW, KB, HSN), NCI P30CA177558 (CH), NIDDK R01DK075787 (JNH), NCI UM1CA186107 (PK), European Regional Development Fund (Project No. 2014-2020.4.01.15-0012) and the European Union's Horizon 2020 research and innovation program under grant agreements No 692065 (TL, RM, AS) and 692145 (RM), Estonian Research Council grant PRG1076 (AS), Horizon 2020 innovation grant ERIN grant EU952516 (AS), Horizon Europe NESTOR grant 101120075 (AS), NICHD R01HD065029 (RS), Estonian Ministry of Education and Research (grant IUT34-16 to TL), *NICHD R01HD057450 (MU), NICHD R01HD100630(MU, RL, MGH, CW),* NICHD P50HD044405 (AD), NICHD R01HD057223 (AD), R01HD085227 (MGH, AD) and R01 HD100812 (AD) from the Eunice Kennedy Shriver National Institute of Child Health and Human Development, deCode Genetics (GT, UT, KS, US*), NHMRC Ideas Grant 2003629 and DoH Western Australia Merit Award 1186046 (BHM),* SCGOPHCG RAC 2015-16/034 (SGW, BGAS), 2016-17/018 (BGAS), NIHR BRC, Wellcome Trust, MRC (TDS), Eris M. Field Chair in Diabetes Research (MOG), NIDDK P30 DK063491 (MOG), NIDDK U01DK094431, U01DK048381 (DE), NICHD U10HD38992 (RL), Estonian Ministry of Education and Research (grant IUT34-16), Enterprise Estonia (grant EU48695); the EU-FP7 Marie Curie Industry-Academia Partnerships and Pathways (IAPP, grant SARM, EU324509 to AS), Wellcome (090532, 098381, 203141); European Commission (ENGAGE: HEALTH-F4-2007-201413 to MIM), MRC G0802782, MR/M012638/1 (SF), NIH R01 HD057223 (AD), R01 HD085227 (AD, MGH), R01 HD100812 (AD), Li Ka Shing Foundation, WT-SSI/John Fell Funds, NIHR Biomedical Research Centre, Oxford, Widenlife and NICHD 5P50HD028138-27 (CML), NICHD R01HD065029, ADA 1-10-CT-57, Harvard Clinical and Translational Science Center, from the National Center for Research Resources 1UL1 RR025758 (CKW). Novo Nordisk Foundation Data Science Investigator grant NNF20OC0062294 (TK). Geisinger's MyCode Community Health Initiative (MyCode): We thank all the participants of the MyCode Study. We thank the members of the Geisinger-Regeneron DiscovEHR Collaboration who have been critical in the generation of the genetic data used in this study. The funders had no role in study design, data collection and analysis, decision to publish, or preparation of the manuscript.

Address Changes

Amber DeVries, Spyre Therapeutics, Waltham, MA 02453

Mark I. McCarthy, Genentech, 1 DNA Way, South San Francisco, CA 94080

Lea Davis, Department of Medicine, Division of Data-Driven and Digital Medicine
Icahn School of Medicine at Mount Sinai, New York, NY 10029

Ethics statements

All research involving human participants has been approved by the authors' Institutional Review Board (IRB) or an equivalent committee, and all clinical investigation was conducted according to the principles expressed in the Declaration of Helsinki. Written informed consent was obtained from all participants.

The Boston cohort was approved by the Partners IRB (# 2002P001924 and 2012P002417) and the University of Utah IRB (IRB_00076659). The deCODE cohort was approved by the National Bioethics Committee of Iceland (VSN 03–007), which was conducted in agreement with conditions issued by the Data Protection Authority of Iceland. Personal identities of the participants' data and biological samples were encrypted by a third-party system (Identity Protection System), approved and monitored by the Data Protection Authority.

The UK cohort was approved by the Parkside Health Authority (Now—NHS Health Research Authority, NRES Committee—West London & GTAC, UK, London, UK) under EC2359 "The Molecular Genetics of Polycystic Ovaries."

The Rotterdam PCOS cohort, was approved by institutional review board (Medical Ethics Committee) of the Erasmus Medical Center (04-263).
Controls from the Lifelines Cohort Study have been approved by the UMCG Medical ethical committee under number 2007/152.

The Chicago PCOS cohort was approved by the Northwestern IRB (#STU00008096). The control subjects from the NUgene study were approved by the Northwestern IRB (# STU00010003).

The Estonia cohort was approved by the Research Ethics Committee of the University of Tartu approved the study (198T-18).

The Western Australian PCOS study was approved by the SCGOPHCG Human Research Ethics Committee (RGS0000001467) and controls by HRA North West – Liverpool East Research Ethics Committee (19/NW/0187; TwinsUK).

The Nurses' Health Study (NHS I and II) was approved by the Partners Human Research Committee (#1999-P-011114).

Patients and control subjects in FinnGen provided informed consent for biobank research, based on the Finnish Biobank Act. Alternatively, older research cohorts, collected prior the start of FinnGen (in August 2017), were collected based on study-specific consents and later transferred to the Finnish biobanks after approval by the National Supervisory Authority for Welfare and Health, Fimea. Recruitment procedures followed the biobank protocols approved by Fimea. The Coordinating Ethics Committee of the Hospital District of Helsinki and Uusimaa (HUS) approved the FinnGen study protocol (Nr HUS/990/2017). The FinnGen study was approved by Finnish Institute for Health and Welfare (permit numbers: THL/2031/6.02.00/2017, THL/1101/5.05.00/2017, THL/341/6.02.00/2018, THL/2222/6.02.00/2018, THL/283/6.02.00/2019, THL/1721/5.05.00/2019, THL/1524/5.05.00/2020, and THL/2364/14.02/2020); Digital and population data service

agency (permit numbers: VRK43431/2017-3, VRK/6909/2018-3, VRK/4415/2019-3); the Social Insurance Institution (permit numbers: KELA 58/522/2017, KELA 131/522/2018, KELA 70/522/2019, KELA 98/522/2019, KELA 138/522/2019, KELA 2/522/2020, KELA 16/522/2020); and Statistics Finland (permit numbers: TK-53- 1041-17 and TK-53-90-20). The Biobank access decisions for FinnGen samples and data utilized in the FinnGen Data Freeze 6 include: THL Biobank BB2017_55, BB2017_111, BB2018_19, BB_2018_34, BB_2018_67, BB2018_71, BB2019_7, BB2019_8, BB2019_26, BB2020_1, Finnish Red Cross Blood Service Biobank 7.12.2017, Helsinki Biobank HUS/359/2017, Auria Biobank AB17-5154, Biobank Borealis of Northern Finland_2017_1013, Biobank of Eastern Finland 1186/2018, Finnish Clinical Biobank Tampere MH0004, Central Finland Biobank 1-2017, and Terveystalo Biobank STB 2018001.

Analyses in the EstBB were carried out under ethical approval 1.1-12/624 from the Estonian Committee on Bioethics and Human Research and data release N05 from the EstBB.

Cedars Sinai -The study was approved by the institutional review boards of the recruiting centers and Cedars-Sinai Medical Center (CSMC). Written informed consent was obtained from all participants.

BioVU – approved by the Institutional Review Board at Vanderbilt University (#160279).

At enrolment, Women's Genome Health Study (WGHS) participants consented to ongoing analyses linking blood-derived observations with clinical measures collected at baseline and throughout observation. All analysis in the WGHS had been approved by the institutional review board of Brigham and Women's Hospital, Boston, MA.

The Michigan Genomics Initiative (MGI) adhered to the principles of the Declaration of Helsinki. Consent forms and study protocols for MGI participants received approval from the Institutional Review Board of the University of Michigan Medical School (IRB IDs: HUM00071298, HUM00099197, HUM00151834, HUM00156162, HUM00164162, HUM00143789, HUM00167679, HUM00041845, HUM00044507, HUM00176147, HUM00148297, HUM00141565, and HUM00097962).

The Genes & Health study was approved by the London South East NRES Committee of the Health Research Authority (14/LO/1240).

BioMe was approved by the Icahn School of Medicine at Mount Sinai's Institutional Review Board (23-00583). All participants provided written informed consent.

The Danish Blood Donor Study (DBDS): Ethics committee approval NVK-1700407, data protection agency P-2019-99. Repro: Ethics committee approval NVK-1805807, data protection agency P-2019-49.

All participants provided informed consent to participate in the original MyCode Community Health Initiative as approved by the Geisinger Institutional Review Board. The project described in this paper was reviewed and determined to be not human subjects research by the Geisinger Institutional Review Board (IRB#: #2017-158).

Supplementary References

1. Chang S, Dunaif A. Diagnosis of Polycystic Ovary Syndrome: Which Criteria to Use and When? *Endocrinol Metab Clin North Am* 2021;50:11-23.

2. Day FR, Hinds DA, Tung JY, Stolk L, Styrkarsdottir U, Saxena R, Bjonnes A, Broer L, Dunger DB, Halldorsson BV, Lawlor DA, Laval G, Mathieson I, McCardle WL, Louwers Y, Meun C, Ring S, Scott RA, Sulem P, Uitterlinden AG, Wareham NJ, Thorsteinsdottir U, Welt C, Stefansson K, Laven JS, Ong KK, Perry JR. Causal mechanisms and balancing selection inferred from genetic associations with polycystic ovary syndrome. *Nat Commun* 2015;6:8464.

3. Ferriman D, Gallwey JD. Clinical assessment of body hair growth in women. *J Clin Endocrinol Metab* 1961;21:1440-7.

4. Rotterdam EA-SPcwg. Revised 2003 consensus on diagnostic criteria and long-term health risks related to polycystic ovary syndrome (PCOS). *Hum Reprod* 2004;19:41-7.

5. Zhu Z, Zhang F, Hu H, Bakshi A, Robinson MR, Powell JE, Montgomery GW, Goddard ME, Wray NR, Visscher PM, Yang J. Integration of summary data from GWAS and eQTL studies predicts complex trait gene targets. *Nat Genet* 2016;48:481-7.

6. Perry JR, Hsu YH, Chasman DI, Johnson AD, Elks C, Albrecht E, Andrulis IL, Beesley J, Berenson GS, Bergmann S, Bojesen SE, Bolla MK, Brown J, Buring JE, Campbell H, Chang-Claude J, Chenevix-Trench G, Corre T, Couch FJ, Cox A, Czene K, D'Adamo A P, Davies G, Deary IJ, Dennis J, Easton DF, Engelhardt EG, Eriksson JG, Esko T, Fasching PA, Figueroa JD, Flyger H, Fraser A, Garcia-Closas M, Gasparini P, Gieger C, Giles G, Guenel P, Hagg S, Hall P, Hayward C, Hopper J, Ingelsson E, kConFab i, Kardia SL, Kasiman K, Knight JA, Lahti J, Lawlor DA, Magnusson PK, Margolin S, Marsh JA, Metspalu A, Olson JE, Pennell CE, Polasek O, Rahman I, Ridker PM, Robino A, Rudan I, Rudolph A, Salumets A, Schmidt MK, Schoemaker MJ, Smith EN, Smith JA, Southey M, Stockl D, Swerdlow AJ, Thompson DJ, Truong T, Ulivi S, Waldenberger M, Wang Q, Wild S, Wilson JF, Wright AF, Zgaga L, ReproGen C, Ong KK, Murabito JM, Karasik D, Murray A. DNA mismatch repair gene MSH6 implicated in determining age at natural menopause. *Hum Molec Genet* 2014;23:2490-7.

7. Zhang J, Dutta D, Kottgen A, Tin A, Schlosser P, Grams ME, Harvey B, CKDGen Consortium, Yu B, Boerwinkle E, Coresh J, Chatterjee N. Plasma proteome analysis in individuals of European and African ancestry identify cis-pQTLs and models for proteome-wide association studies. *Nat Genet* 2022;54:593-602.

8. GTex Consortium, Battle A, Brown CD, Engelhardt BE, Montgomery SB. Genetic effects on gene expression across human tissues. *Nature* 2017;550:204-13.

9. Khera AV, Chaffin M, Aragam KG, Haas ME, Roselli C, Choi SH, Natarajan P, Lander ES, Lubitz SA, Ellinor PT, Kathiresan S. Genome-wide polygenic scores for common diseases identify individuals with risk equivalent to monogenic mutations. *Nat Genet* 2018;50:1219-24.

10. Udler MS, Kim J, von Grotthuss M, Bonas-Guarch S, Cole JB, Chiou J, Christopher DAoboM, the I, Boehnke M, Laakso M, Atzmon G, Glaser B, Mercader JM, Gaulton K, Flannick J, Getz G, Florez JC. Type 2 diabetes genetic loci informed by multi-trait associations point to disease mechanisms and subtypes: A soft clustering analysis. *PLoS Med* 2018;15:e1002654.

11. Eastwood SV, Mathur R, Atkinson M, Brophy S, Sudlow C, Flaig R, de Lusignan S, Allen N, Chaturvedi N. Algorithms for the Capture and Adjudication of Prevalent and Incident Diabetes in UK Biobank. *PLoS One* 2016;11:e0162388.

12. Smith DJ, Nicholl BI, Cullen B, Martin D, Ul-Haq Z, Evans J, Gill JM, Roberts B, Gallacher J, Mackay D, Hotopf M, Deary I, Craddock N, Pell JP. Prevalence and characteristics of

probable major depression and bipolar disorder within UK biobank: cross-sectional study of 172,751 participants. *PLoS One* 2013;8:e75362

13. Shi Y, Zhao H, Shi Y, Cao Y, Yang D, Li Z, Zhang B, Liang X, Li T, Chen J, Shen J, Zhao J, You L, Gao X, Zhu D, Zhao X, Yan Y, Qin Y, Li W, Yan J, Wang Q, Zhao J, Geng L, Ma J, Zhao Y, He G, Zhang A, Zou S, Yang A, Liu J, Li W, Li B, Wan C, Qin Y, Shi J, Yang J, Jiang H, Xu JE, Qi X, Sun Y, Zhang Y, Hao C, Ju X, Zhao D, Ren CE, Li X, Zhang W, Zhang Y, Zhang J, Wu D, Zhang C, He L, Chen ZJ. Genome-wide association study identifies eight new risk loci for polycystic ovary syndrome. *Nat Genet.* 2012 Sep;44(9):1020-5

14. Semple RK, Savage DB, Cochran EK, Gorden P, O'Rahilly S. Genetic Syndromes of Severe Insulin Resistance. *Endocrine Reviews*, 2011;32(4):498–514.

15. Urbanek M, Legro RS, Driscoll DA, Azziz R, Ehrmann DA, Norman RJ, Strauss JF 3rd, Spielman RS, Dunaif A. Thirty-seven candidate genes for polycystic ovary syndrome: strongest evidence for linkage is with follistatin. Proc Natl Acad Sci U S A. 1999 Jul 20;96(15):8573-8.

16. Oh HS, Rutledge J, Nachun D, Pálovics R, Abiose O, Moran-Losada P, Channappa D, Urey DY, Kim K, Sung YJ, Wang L, Timsina J, Western D, Liu M, Kohlfeld P, Budde J, Wilson EN, Guen Y, Maurer TM, Haney M, Yang AC, He Z, Greicius MD, Andreasson KI, Sathyan S, Weiss EF, Milman S, Barzilai N, Cruchaga C, Wagner AD, Mormino E, Lehallier B, Henderson VW, Longo FM, Montgomery SB, Wyss-Coray T. Organ aging signatures in the plasma proteome track health and disease. *Nature.* 2023 Dec;624(7990):164-172.

17. Hemani G, Tilling K, Davey Smith G. Orienting the causal relationship between imprecisely measured traits using GWAS summary data. *PLoS Genet.* 2017 Nov 17;13(11):e1007081.

18. Day F, Karaderi T, Jones MR, Meun C, He C, Drong A, Kraft P, Lin N, Huang H, Broer L, Magi R, Saxena R, Laisk T, Urbanek M, Hayes MG, Thorleifsson G, Fernandez-Tajes J, Mahajan A, Mullin BH, Stuckey BGA, Spector TD, Wilson SG, Goodarzi MO, Davis L, Obermayer-Pietsch B, Uitterlinden AG, Anttila V, Neale BM, Jarvelin MR, Fauser B, Kowalska I, Visser JA, Andersen M, Ong K, Stener-Victorin E, Ehrmann D, Legro RS, Salumets A, McCarthy MI, Morin-Papunen L, Thorsteinsdottir U, Stefansson K, andMe Research T, Styrkarsdottir U, Perry JRB, Dunaif A, Laven J, Franks S, Lindgren CM, Welt CK. Large-scale genome-wide meta-analysis of polycystic ovary syndrome suggests shared genetic architecture for different diagnosis criteria. *PLoS Genet* 2018;14:e1007813.

19. Sijtsma A, Rienks J, van der Harst P, Navis G, Rosmalen JGM, Dotinga A. Cohort prrofile update: Lifelines, a three-generation cohort study and biobank. *Int J Epidemiol* 2022;51:e295-e302.
