# Supplementary material for: Genomic analyses implicate hormonal and metabolic dysregulation in polycystic ovary syndrome
Source: Nat Genet. 2026 Apr 23;58(5):1040–50. doi: 10.1038/s41588-026-02543-9 (PMC13175888; doi:10.1038/s41588-026-02543-9)
Supplement: Supplementary file 2 — Reporting Summary [file 41588_2026_2543_MOESM2_ESM.pdf]

nature portfolio

Corresponding author(s): Felix Day

Last updated by author(s): Apr 3, 2025

Reporting Summary

Nature Portfolio wishes to improve the reproducibility of the work that we publish. This form provides structure for consistency and transparency in reporting. For further information on Nature Portfolio policies, see our Editorial Policies and the Editorial Policy Checklist.

Statistics

For all statistical analyses, confirm that the following items are present in the figure legend, table legend, main text, or Methods section.

n/a	Confirmed	
☐	☒	The exact sample size (*n*) for each experimental group/condition, given as a discrete number and unit of measurement
☐	☒	A statement on whether measurements were taken from distinct samples or whether the same sample was measured repeatedly
☐	☒	The statistical test(s) used AND whether they are one- or two-sided<br>*Only common tests should be described solely by name; describe more complex techniques in the Methods section.*
☐	☒	A description of all covariates tested
☐	☒	A description of any assumptions or corrections, such as tests of normality and adjustment for multiple comparisons
☐	☒	A full description of the statistical parameters including central tendency (e.g. means) or other basic estimates (e.g. regression coefficient) AND variation (e.g. standard deviation) or associated estimates of uncertainty (e.g. confidence intervals)
☐	☒	For null hypothesis testing, the test statistic (e.g. *F*, *t*, *r*) with confidence intervals, effect sizes, degrees of freedom and *P* value noted<br>*Give P values as exact values whenever suitable.*
☐	☒	For Bayesian analysis, information on the choice of priors and Markov chain Monte Carlo settings
☐	☒	For hierarchical and complex designs, identification of the appropriate level for tests and full reporting of outcomes
☐	☒	Estimates of effect sizes (e.g. Cohen's *d*, Pearson's *r*), indicating how they were calculated

Our web collection on statistics for biologists contains articles on many of the points above.

Software and code

Policy information about availability of computer code

Data collection	No software was used.
Data analysis	Central quality control (QC) was performed using the EasyQC pipeline (version 9.2). A fixed-effect, inverse-weighted-variance meta-analysis approach was used with the collected summary statistics from the individual studies, using either GWAMA version is 1_2_6) or METAL (version released on 2011-03-25). Identified variants were annotated using FUMA (v1.5.2). Forest plots for comparing the effect sizes across the strata in the meta-analysis were made using the ggplot2 package in R (version 4.3.1).<br><br>Fine-mapping was performed using the shotgun stochastic search method as performed in FINEMAP. Functional mapping and annotation of GWAS was performed with FUMA (v1.5.2), and further annotation of the association results with PhenoScanner (date accessed 25 March 2022).<br><br>The GWAS-to-Genes pipeline, incorporating eQTL colocalisation from both SMR-HEIDI (Version 0.68) and the “coloc” package, was used to highlight likely causal genes at each of the identified signals. We used the PRS-CS software to calculate a polygenic risk score (PRS) for PCOS.<br><br>In the same UK Biobank sample, we replicated these analyses using another PRS tool, LDpred-2, which employs Bayesian shrinkage model. PRS for PCOS were also calculated in the Copenhagen Hospital Biobank using LDPred2. GWAS summary statistics were pre-processed with MungeSumStats. The AER R package (v1.2.10) was used to determine whether there was equi-, under-, or over-dispersion. Lastly, using the Danish IVF registry, data models were fit using glmmTMB (/services/tools/R/4.0.0/R_PACKAGES.txt:glmmTMB “1.1.5”).

For manuscripts utilizing custom algorithms or software that are central to the research but not yet described in published literature, software must be made available to editors and reviewers. We strongly encourage code deposition in a community repository (e.g. GitHub). See the Nature Portfolio guidelines for submitting code & software for further information.

Data

Policy information about availability of data

All manuscripts must include a data availability statement. This statement should provide the following information, where applicable:

- Accession codes, unique identifiers, or web links for publicly available datasets
- A description of any restrictions on data availability
- For clinical datasets or third party data, please ensure that the statement adheres to our policy

Cohorts should be contacted individually for access to their raw data. UK Biobank data are available on application (https://ams.ukbiobank.ac.uk/ams/). Access to the two strata summary statistics can be found at the DOI in the data access section of the paper.

We used the NCBI RefSeq gene map for GRCh37 which is available via http://hgdownload.soe.ucsc.edu/goldenPath/hg19/database/. GTEx eQTL data was used (V7) and is available via https://gtexportal.org.

Research involving human participants, their data, or biological material

Policy information about studies with human participants or human data. See also policy information about sex, gender (identity/presentation), and sexual orientation and race, ethnicity and racism.

Reporting on sex and gender

All analysis are disaggregated by sex.
All included studies had ethics approval and collected informed written consent.

Reporting on race, ethnicity, or other socially relevant groupings

Study samples were defined within each study that contributed to the meta-analysis, usually by position based on genetic principle components. As these were done at a study level, we have used the geographical based ancestry terminology.

Population characteristics

Described in Supplementary Table 1 "Cohort descriptives"

Recruitment

This study is a meta-analysis, combining data from different sources across the world. Recruitment varied across the different included studies.

Ethics oversight

UK Biobank data has approval from the North West Multi-centre Research Ethics Committee (MREC) as a Research Tissue Bank (RTB). 23andMe research participants provided informed consent and volunteered to participate in the research online under a protocol approved by the external AAHRPP-accredited IRB, Ethical & Independent (E&I) Review Services. For each of the other individual studies that contributed data, ethical approval was given by the relevant boards.

The Boston cohort was approved by the Partners IRB (# 2002P001924 and 2012P002417) and the University of Utah IRB (IRB_00076659). The deCODE cohort was approved by the National Bioethics Committee of Iceland (VSN 03–007), which was conducted in agreement with conditions issued by the Data Protection Authority of Iceland. Personal identities of the participants' data and biological samples were encrypted by a third-party system (Identity Protection System), approved and monitored by the Data Protection Authority.

The UK cohort was approved by the Parkside Health Authority (Now—NHS Health Research Authority, NRES Committee—West London & GTAC, UK, London, UK) under EC2359 "The Molecular Genetics of Polycystic Ovaries."

The Rotterdam PCOS cohort, was approved by institutional review board (Medical Ethics Committee) of the Erasmus Medical Center (04-263). Controls from the Lifelines Cohort Study have been approved by the UMCG Medical ethical committee under number 2007/152.

The Chicago PCOS cohort was approved by the Northwestern IRB (#STU00008096). The control subjects from the NUgene study were approved by the Northwestern IRB (# STU00010003).

The Estonia cohort was approved by the Research Ethics Committee of the University of Tartu approved the study (198T-18).

The Western Australian PCOS study was approved by the SCGOPHCG Human Research Ethics Committee (RGS0000001467) and controls by HRA North West – Liverpool East Research Ethics Committee (19/NW/0187; TwinsUK).

The Nurses' Health Study (NHS I and II) was approved by the Partners Human Research Committee (#1999-P-011114).

Patients and control subjects in FinnGen provided informed consent for biobank research, based on the Finnish Biobank Act. Alternatively, older research cohorts, collected prior the start of FinnGen (in August 2017), were collected based on study-specific consents and later transferred to the Finnish biobanks after approval by the National Supervisory Authority for Welfare and Health, Fimea. Recruitment procedures followed the biobank protocols approved by Fimea. The Coordinating Ethics Committee of the Hospital District of Helsinki and Uusimaa (HUS) approved the FinnGen study protocol (Nr HUS/990/2017). The FinnGen study was approved by Finnish Institute for Health and Welfare (permit numbers: THL/2031/6.02.00/2017, THL/1101/5.05.00/2017, THL/341/6.02.00/2018, THL/2222/6.02.00/2018, THL/283/6.02.00/2019, THL/1721/5.05.00/2019, THL/1524/5.05.00/2020, and THL/2364/14.02/2020); Digital and population data service agency (permit numbers: VRK43431/2017-3, VRK/6909/2018-3, VRK/4415/2019-3); the Social Insurance Institution (permit numbers: KELA 58/522/2017, KELA 131/522/2018, KELA 70/522/2019, KELA 98/522/2019, KELA 138/522/2019, KELA 2/522/2020, KELA 16/522/2020); and Statistics Finland (permit numbers: TK-53- 1041-17 and TK-53-90-20). The Biobank access decisions for

FinnGen samples and data utilized in the FinnGen Data Freeze 6 include: THL Biobank BB2017_55, BB2017_111, BB2018_19, BB_2018_34, BB_2018_67, BB2018_71, BB2019_7, BB2019_8, BB2019_26, BB2020_1, Finnish Red Cross Blood Service Biobank 7.12.2017, Helsinki Biobank HUS/359/2017, Auria Biobank AB17-5154, Biobank Borealis of Northern Finland_2017_1013, Biobank of Eastern Finland 1186/2018, Finnish Clinical Biobank Tampere MH0004, Central Finland Biobank 1-2017, and Terveystalo Biobank STB 2018001.

Analyses in the EstBB were carried out under ethical approval 1.1-12/624 from the Estonian Committee on Bioethics and Human Research and data release N05 from the EstBB.

Cedars Sinai -The study was approved by the institutional review boards of the recruiting centers and Cedars-Sinai Medical Center (CSMC). Written informed consent was obtained from all participants.

BioVU – approved by the Institutional Review Board at Vanderbilt University (#160279).

At enrolment, Women's Genome Health Study (WGHS) participants consented to ongoing analyses linking blood-derived observations with clinical measures collected at baseline and throughout observation. All analysis in the WGHS had been approved by the institutional review board of Brigham and Women’s Hospital, Boston, MA.

The Michigan Genomics Initiative (MGI) adhered to the principles of the Declaration of Helsinki. Consent forms and study protocols for MGI participants received approval from the Institutional Review Board of the University of Michigan Medical School (IRB IDs: HUM00071298, HUM00099197, HUM00151834, HUM00156162, HUM00164162, HUM00143789, HUM00167679, HUM00041845, HUM00044507, HUM00176147, HUM00148297, HUM00141565, and HUM00097962).

The Genes & Health study was approved by the London South East NRES Committee of the Health Research Authority (14/LO/1240).

BioMe was approved by the Icahn School of Medicine at Mount Sinai's Institutional Review Board (23-00583). All participants provided written informed consent.

The Danish Blood Donor Study (DBDS): Ethics committee approval NVK-1700407, data protection agency P-2019-99. Repro: Ethics committee approval NVK-1805807, data protection agency P-2019-49.

All participants provided informed consent to participate in the original MyCode Community Health Initiative as approved by the Geisinger Institutional Review Board. The project described in this paper was reviewed and determined to be not human subjects research by the Geisinger Institutional Review Board (IRB#: #2017-158).

Note that full information on the approval of the study protocol must also be provided in the manuscript.

Field-specific reporting

Please select the one below that is the best fit for your research. If you are not sure, read the appropriate sections before making your selection.

☒ Life sciences ☐ Behavioural & social sciences ☐ Ecological, evolutionary & environmental sciences

For a reference copy of the document with all sections, see nature.com/documents/nr-reporting-summary-flat.pdf

Life sciences study design

All studies must disclose on these points even when the disclosure is negative.

Sample size	This study included summary statistics of our previous meta-analysis including 10,074 cases and 103,164 controls of European ancestry (described in detail in Day et al. 2019). An additional 13 cohorts were included for the current GWAS meta-analysis, resulting in 21,570 cases and 523,971 controls. The newly added cases included all-ancestries, of which 87% of the cases were from European origin, 10% from East-Asian origin and the remaining being either from Hispanic or African-American ancestry.
Data exclusions	Individuals failing standard genotyping quality control parameters defined in the individual studies or missing genotype, phenotype, or covariate data were excluded from analysis. This decision was made prior to performing any downstream analysis.
Replication	We conducted our analysis using a sufficiently powered dataset. Additionally, no suitable dataset was available for replication. However, we did replicate our PRS in the Biobank, testing for the presence of PCOS.
Randomization	Participants were not allocated into experimental groups. The principle exposure in this study is naturally occurring genetic variants, meaning that we were unable to randomize the individuals in the study.
Blinding	Blinding is by design not possible in this study, as it is a genome wide association study of common and rare genetic variants and not a randomized study.

Reporting for specific materials, systems and methods

We require information from authors about some types of materials, experimental systems and methods used in many studies. Here, indicate whether each material, system or method listed is relevant to your study. If you are not sure if a list item applies to your research, read the appropriate section before selecting a response.

Materials & experimental systems

n/a	Involved in the study
☒	☐ Antibodies
☒	☐ Eukaryotic cell lines
☒	☐ Palaeontology and archaeology
☒	☐ Animals and other organisms
☒	☐ Clinical data
☒	☐ Dual use research of concern
☒	☐ Plants

Methods

n/a	Involved in the study
☒	☐ ChIP-seq
☒	☐ Flow cytometry
☒	☐ MRI-based neuroimaging

Plants

Seed stocks	N/A
Novel plant genotypes	N/A
Authentication	N/A
